# Building a Successful Massive Open Online Course About Multiple Sclerosis: A Process Description

**DOI:** 10.2196/16687

**Published:** 2020-07-29

**Authors:** Suzi B Claflin, Rachael Gates, Maree Maher, Bruce V Taylor

**Affiliations:** 1 Menzies Institute for Medical Research University of Tasmania Hobart Australia; 2 Wicking Dementia Research and Education Centre University of Tasmania Hobart Australia; 3 Multiple Sclerosis Limited Blackburn Australia

**Keywords:** multiple sclerosis, health education

## Abstract

**Background:**

Over the course of a year, we developed and tested a 6-week massive open online course (MOOC) on multiple sclerosis (MS) in consultation with the MS community. The course targeted the MS community and interested laypeople and was titled *Understanding MS*. The primary purpose of the course was to improve MS knowledge, health literacy, and resilience among participants. The final version of the MOOC made available for open enrollment was ranked first among all MOOCs released in 2019 (n>2400) based on participant reviews.

**Objective:**

The aim of this study was to present a detailed description and assessment of the development process of the *Understanding MS* MOOC.

**Methods:**

The development process included a course development focus group; the creation of more than 50 content videos and related text, quizzes, activities, and discussion prompts; the creation of original images and animations; a pilot study; and collaborations with people living with MS, MS nurses, allied health care practitioners, and neurologists and researchers from 4 universities.

**Results:**

Overall, the process was efficient and effective. With a few small changes, we recommend this approach to those seeking to develop a similar course. This process led to the development of a highly reviewed MOOC with excellent user satisfaction.

**Conclusions:**

We identified 5 key lessons from this process: (1) community support is essential, (2) stakeholder involvement improves content quality, (3) plan for research from the beginning, (4) coordination between the academic lead and project manager team ensures a consistent voice, and (5) a network of collaborators is a key resource.

## Introduction

### Background

Massive open online courses (MOOCs) have the potential to extend the classroom to people who cannot access traditional learning environments by capitalizing on the widespread availability of the internet [1]. The defining characteristics of MOOCs are that they are freely available and have virtually unlimited enrollment capacity. These features make MOOCs versatile; they can be used to present information on a range of topics to an array of different audience.

Over the past decade, there has been a rapid increase in the development of MOOCs. Class Central, the leading MOOC aggregator site, currently lists more than 13,000 MOOCs from more than 900 universities [2]. However, there are significant challenges in MOOC development and implementation, including the cost and time involved [3,4], with universities spending US $200,000 or more per course [4]. These challenges suggest that it is worth evaluating the approaches used to create MOOCs to identify strategies that are cost-effective and result in successful courses.

Furthermore, little is known about the impact of MOOCs on learning outcomes. Addressing this knowledge gap requires meaningful comparisons of many courses [5]. These comparisons require an understanding of the similarities and differences in course development and content. However, little research has been done describing the process of developing an MOOC in detail [6,7]. Here, we present a detailed description and assessment of our work—developing an MOOC on multiple sclerosis (MS) for the MS community and interested laypeople—including all tools and materials that we used or produced, in the hope that it assists others in the creation of similar content and allows for more meaningful comparisons between courses.

MS is a disease of the central nervous system that affects approximately 2.3 million people worldwide [8]. People living with MS often experience mobility and fatigue-related symptoms that can interfere with their ability to access health care [9]. Consequently, several digital and remote communication disease management technologies have been developed for people living with MS [10]. MS symptoms may also impede the ability of people living with MS to access traditional educational offerings (eg, in-person courses), making it difficult for them to travel to a particular meeting place or maintain active participation over long sessions. Therefore, an MOOC may be a more appropriate vehicle for knowledge translation for people living with MS and other members of the MS community (eg, family and friends, caregivers, and health care providers), particularly for those who may feel isolated by living with a rare condition.

### Objectives

Here, we describe and assess our experience in developing a successful 6-week English-language MOOC about MS. The first enrollment ran from April to June 2019 and the second ran from September to November 2019, with more than 8000 people enrolling and an average completion rate of 47% (SB Claflin, unpublished data, 2019), far exceeding the average for MOOCs, which fluctuates between 5% and 15% [11,12]. On the basis of participant reviews from these 2 enrollments, the course was ranked first among more than 2400 MOOCs released in 2019 and third among all health and medicine MOOCs globally by the leading online course aggregator site, Class Central [13,14]. In this paper, we described the process we used to develop the *Understanding MS* MOOC in detail, highlighting the strengths and weaknesses of our approach and making recommendations for the development of similar interventions.

## Methods

### Ethics

The work conducted for this project was approved by the Social Science Human Research Ethics Committee at the University of Tasmania (UTAS), including the focus group (H0017241) and a pilot study (H0017778).

### Funding and Costs

The Understanding MS MOOC was collaboratively developed by the MS Flagship at the Menzies Institute for Medical Research (Menzies) at UTAS and Multiple Sclerosis Limited, a service organization providing resources to the MS communities of New South Wales, Victoria, Tasmania, and the Australian Capital Territory. These organizations cofunded the project, resulting in a total budget of approximately AUD $200,000 (US $139,000). The majority of these funds were used to pay the salary of a full-time postdoctoral research fellow who served as the academic lead of the project (SC) and the secondment of a MOOC project manager (RG) from the Wicking Center for Dementia Research and Education (WDREC). A small amount of funding was used to develop some of the course animations and to pay for travel costs and video transcription.

The remainder of the costs related to course development were in-kind, provided in either time or materials. The area experts who presented the course videos did it for free. The UTAS employs a videographer who filmed all but a few of the videos. These videos were primarily shot in studios that were generously offered at no cost by the local Australian Broadcasting Corporation and the University of Melbourne. All video editing was performed by the UTAS staff. All images were reproduced for free with permission from the original source or developed by the UTAS staff. A few animations were also produced in-house. Finally, the course was hosted on a custom UTAS-built MOOC platform, which was originally constructed to host MOOCs produced by the WDREC [5].

### Management Structure

The development of the MOOC was primarily overseen by 2 full-time employees: the academic lead and the project manager. The academic lead was responsible for the development of course content, including syllabus, video outlines, text, quizzes, discussion prompts, and activities. The academic lead was also responsible for video presentations, both presenting by herself and liaising with other area experts. In addition, she was responsible for developing the research projects surrounding the MOOC and leading the focus group, pilot study, and development of the MS Knowledge Assessment Scale. The project manager was responsible for coordinating and overseeing video production (including editing and transcription; scheduling filming; and liaising with external contractors, such as the video animator), establishing branding and style guidelines for the course, liaising on technical requirements for the web-based learning management system, coordinating digital marketing strategies for the course, coordinating and implementing communication processes for participant engagement and retention, and general project planning and administration tasks. The academic lead and project manager worked closely together, usually meeting for the project several times per week and sometimes as often as several times per day.

The project was also overseen by an advisory group that met fortnightly throughout the project. The group included a clinician-researcher who served as the academic lead’s direct supervisor (BT); Menzies MS Flagship project manager; Menzies MS Flagship communications officer; Menzies business manager; MS researchers, including an epidemiologist, a neuroscientist, and a health economist; a web-based learning and systems support manager; and a liaison from Multiple Sclerosis Limited (MM). The group contained a wide range of MS community expertise, from the lived experience of people living with MS to the clinical expertise of a neurologist and an MS nurse. This group provided feedback on course content and study design, contributed their expertise to course videos, and recruited other area experts. All the members of the advisory group were asked to provide their feedback in preparation for this assessment.

### Core Principles and Development Outline

The advisory group developed 3 core principles that guided our work:

1. *Do no harm*. The course should not frighten participants and should be sensitive to participant anxiety.
2. *Keep it positive*. We used positive language in the course and course-related materials.
3. *Share the journey*. We wanted the course to be inclusive in design, content, and presentation and involve a diverse group of MS community members in all stages of development.

**Figure 1:**
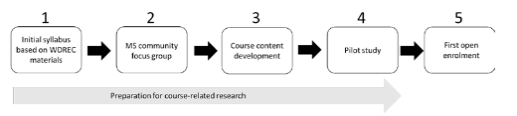
Course development flowchart. MS: multiple sclerosis; WDREC: Wicking Center for Dementia Research and Education.

Course development included 4 distinct steps leading to the implementation of the course (initial syllabus development, focus group, content development, and pilot study) in the first open enrollment. Alongside this work, we carried out preparations for course-related research. The steps of the development process are illustrated in Figure 1.

### Initial Course Syllabus Development

The initial course syllabus was modeled on the work of the WDREC. WDREC previously developed 2 extremely successful MOOCs, Understanding Dementia and Preventing Dementia [15]. The syllabuses for these courses were used as an initial template for the Understanding MS course syllabus. This template was then adapted to fit the MS context. This included adjusting the content to ensure its relevance to people living with MS, as the WDREC courses are primarily taken by caregivers rather than people living with dementia. The initial course syllabus is available in the supplementary materials (Multimedia Appendix 1).

### Focus Group

The main purpose of the focus group was to determine the content and modes of delivery that were most important and/or acceptable to the MS community, particularly people living with MS. The focus group participants were purposefully recruited through existing relationships with Multiple Sclerosis Limited to ensure that the focus group was broadly representative of the larger MS community (eg, representation of multiple MS phenotypes, male and female representation, and a variety of MS community roles). Before participation in the focus group, small group facilitators were given a 1.5-hour facilitator training by an experienced facilitator and a translational researcher. This training covered the protocol of the focus group and facilitation approaches and techniques.

The focus group followed a modified World Café [16] approach:

- Introduction: small group facilitators and MS MOOC team members greeted participants, and the academic lead gave a brief introduction presentation, including an agenda for the day, context and background for the discussion, rationale for developing an online course, and examples of the various modes of delivery available to the team (eg, video types, etc). This was followed by an introduction exercise and a large group discussion of ways of working to establish rules for the small group discussions. After this discussion, a catered morning tea was provided.
- Small group discussions: small group discussions followed the World Café format, except that, because of mobility issues, the participants remained at the same table throughout, and the facilitators moved between tables. Small groups discussed 4 questions, and each question was discussed for 15 to 20 min. Discussion questions are available in the supplementary materials (Multimedia Appendix 2). Discussions were followed by a catered lunch.
- Large group discussion and *dotacracy*: the small group facilitators summarized the main points of the discussions into bullet points, which were posted on a whiteboard and presented to the large group after lunch. Each participant was given a strip of 12 red dot stickers (3 per discussion question). They were told to place the stickers next to the summarized statements that they felt where most important for inclusion in the course. They were allowed to place more than one dot next to a given statement.
- Wrap-up: after voting, the participants were thanked and invited to participate in the course pilot study. The small group facilitators were asked to submit their feedback on the process within a week of the event.

The results of the focus group were analyzed quantitatively using summary statistics and qualitatively using textual analysis. Each group discussion was considered a data item (n=16). All discussion notes were transcribed and assessed using thematic analysis. Any topic mentioned at least twice throughout the data set was considered to be a theme. Any theme mentioned by all 4 groups was considered a key theme. The data collected from the focus group were used to amend the initial syllabus and create the initial course outline, which served as the blueprint for the core course content. The results also informed the style and mode of delivery decisions for course materials.

### Development of Course Materials

The course materials were designed in accordance with our core principles and integrated with the feedback of the focus group participants and the advisory group. The academic lead wrote the first draft of the core course content by elaborating on the course outline, breaking down larger topics into their component pieces, and giving greater detail on each one. The course was structured into 6 modules, with each module comprising several sections. The course outline was adapted into video scripts, with nearly every section containing at least one video, and was distributed to the area expert presenting the information. The area experts were given the opportunity to refine the content of the video with the academic lead before and during filming. These academic videos formed the core content of the course and served as the basis for the rest of the course material.

The academic videos were filmed in 3 formats:

1. Interview: the academic lead sat off-camera interviewing the area expert who was on camera. In the final version, tiles with the interview questions appear between the responses of the expert.
2. Conversational: The area expert and the academic lead were on the camera together, side-by-side. The academic lead interviewed the area expert.
3. Direct-to-camera: the academic lead presented a short (<5 min) scripted lecture on a topic. These videos were later animated.

**Figure 2:**
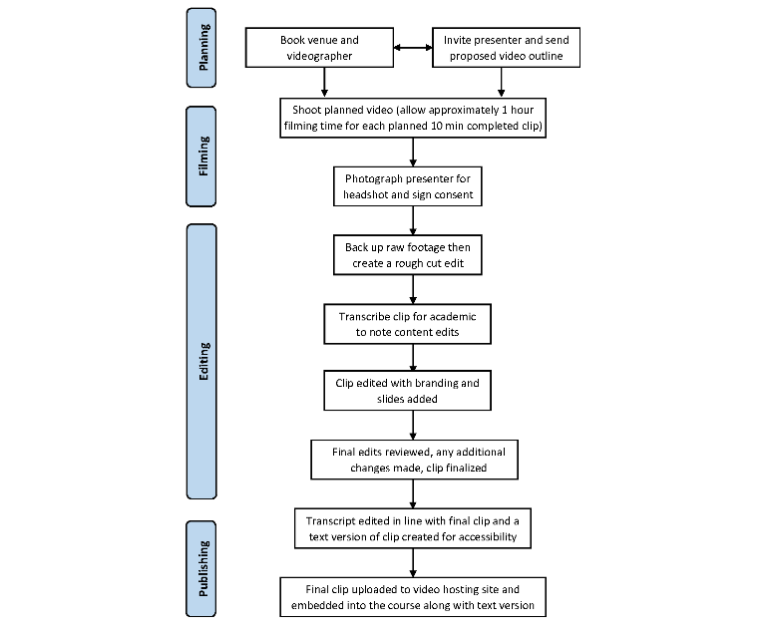
Video production flowchart.

After the videos were shot, they were edited and transcribed so that a text version of all videos could be made available to the course participants. Some videos were animated to illustrate the concepts that they covered. Figure 2 demonstrates the process used to develop video content.

**Figure 3:**
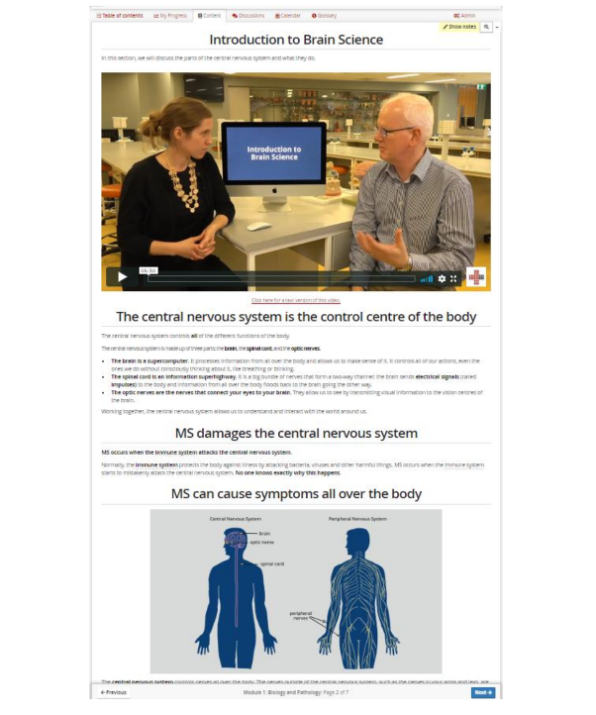
Screen capture showing the layout of a single page of the course.

Text was written to reinforce the main messages of the videos and to add a greater context. The main messages were presented as headers, with more details given in the supporting text. The goal was to ensure that if a participant only read the headers, they would come away with the main points of the section. The text was pitched at a general audience and assumed participants had completed a secondary school science education. Figure 3 shows a screen capture of one of the course pages.

Activities were developed to encourage participant resilience (eg, goal setting) and develop their disease management skillset (eg, symptom tracking). Discussion prompts aimed to inspire reflection about the course content and gather feedback about the activities. End-of-module quizzes aimed to cover the main content of each module, aligning with the module learning objectives. Each quiz was made up of 10 questions, and all questions were multiple choice. Participants had to achieve a score of 70% or higher to move on to the next module, but they could take each quiz as many times as they liked. The correct answers to the quizzes were presented at the beginning of the following module.

In addition, 2 other video series were included in the course: the *community insights* series and *MS perspectives* series. The *MS perspective* series included interviews with people living with MS recruited through pre-existing relationships with Multiple Sclerosis Limited and Menzies. Both were filmed in an interview format. They were each asked the same series of questions, which were related to the core content of the course. The interviews were then spliced together so that the final set of 5 videos showed the experience of multiple people living with MS on a particular subject. The *community insights* series included interviews with other members of the MS community, such as a caregiver and an MS nurse, about their experience working with and/or caring for people living with MS. These videos were placed in the course alongside the core content related to them.

Each module opened with a summary paragraph describing the content, a list of learning objectives, and a list of possible applications for the information presented in the module. The module sections usually began with a video under the section heading, followed by the supporting text. There were 1 to 2 discussion prompts and 1 activity in each module. The final section included a summary paragraph and downloadable summary PDF file, as well as a list of additional resources (mostly links to external websites) and the end-of-module quiz. An example quiz, a list of module activities and discussion prompts, and the finalized course outline are available in the supplementary materials (Multimedia Appendices 3-5). To minimize the burden on course participants, we aimed to keep the time required to complete each module under 2 hours. Due to this, the length of most of our videos was 7 min.

### Pilot Study

The purpose of the pilot study was to gauge MS community members’ satisfaction with the course and to test its technical components. The pilot study was advertised to MS community members via Menzies and Multiple Sclerosis Limited newsletters. People interested in taking part could register their interest on the course’s landing webpage. People who indicated interest were contacted via email and invited to enroll in the pilot study, which began on January 21, 2019. Participants were given access to the course materials in the same fashion as course participants during regular enrollments, with 1 module opening each week accompanied by a reminder email.

Participants were given the option of providing feedback via a web survey at the end of each module and on the overall course. In these surveys, participants were asked to provide quantitative (eg, rating) and qualitative (eg, free text) feedback. Participants rated their satisfaction with each course component (videos, text, images and animations, discussions, quizzes, and technical aspects) on a 5-point Likert scale from very dissatisfied to very satisfied. They were also asked to provide their level of agreement with statements about each course component. The results were analyzed quantitatively using summary statistics (eg, percentage satisfied or in agreement with a given statement) and qualitatively using thematic analysis. An example evaluation survey is available in Multimedia Appendix 6. The results of this study were used to refine the course before the first public iteration, which opened on April 29, 2019.

### Preparation for Course-Related Research

Along with providing community education, research is one of the primary purposes of the Understanding MS MOOC. To prepare for research projects around the first enrollment of the course, the academic lead prepared an implementation evaluation tool and an outcomes evaluation tool, led the development of the MS Knowledge Assessment Scale, and submitted ethics applications for course-related projects. These tools and related research will be described in more detail in future publications.

## Results

In this section, we will assess the methodology we used to develop the Understanding MS MOOC by discussing the strengths and weaknesses of each step in the process.

### Advisory Group and Massive Open Online Course Management Structure

The members of the advisory group reported that the group was effective. As the group contained a cross-section of expertise from all areas of the project (technical, academic, managerial, and clinical) and project stakeholders, as well as key decision makers, decisions were well informed and could be made swiftly. The group was inclusive and respectful of the members’ time. There was an action item list for each meeting, and meetings were not held if there was nothing to discuss. The regular meeting time and buy-in from all members contributed to the group’s success.

Group members reported one notable weakness: the function of the group could be improved by greater budget transparency, which would simplify decision making and purchase approvals.

### Initial Course Syllabus Development

As discussed earlier, access to WDREC materials was a significant advantage when combined with our initial syllabus, as we were able to emulate the structure of successful MOOCs. The only substantial discussion around the initial syllabus was about the module order, particularly the placement of the biology and pathology module. This module is the most technical, and there was a concern that placing it first might discourage participants from completing the course. However, we concluded that placing it first made the most sense, as it provides information that underpins the following modules.

### Focus Group

The small group facilitators agreed that the focus group was a success. They reported that the atmosphere was positive and that the participants enjoyed the process. The focus group had a 100% attendance rate; 21 people were invited and agreed to attend (the demographics of the attendees are provided in Table 1). This was the result of long-standing relationships with the MS flagship business manager, who acted as a community liaison. Furthermore, her initial contact with attendees set a casual and collegial tone for the focus group, which resulted in people arriving relaxed and open. This contributed significantly to the success of the event.

**Table 1:** Demographics of the focus group (n=21). Participants were able to select more than one multiple sclerosis (MS) community role and MS disease course.

Demographics		Values
**Gender, n (%)**		
	Male	6 (29)
	Female	15 (71)
**Education level, n (%)**		
	Secondary school or less	5 (24)
	Occupational certificate or diploma	8 (38)
	Bachelor's degree	4 (19)
	Postgraduate degree	4 (19)
**MS^a^community roles, n (%)**		
	Person with MS	13 (62)
	Family or friend	6 (29)
	Caregiver	3 (14)
	Allied health practitioner	2 (10)
	Advocate	6 (29)
	UTAS^b^ staff member	2 (10)
	Multiple Sclerosis Limited employee	1 (5)
	Volunteer	1 (5)
**Disease course, n (%)**		
	RRMS^c^	9 (82)
	SPMS^d^	1 (9)
	PPMS^e^	0 (0)
	PRMS^f^	0 (0)
	I am not sure	2 (18)
Age (years), mean (SD)		51.14 (11.33)
Disease duration, mean (SD)		13.18 (6.24)
Number of roles in the MS community, mean (SD)		1.62 (0.92)

^a^MS: multiple sclerosis.

^b^UTAS: University of Tasmania.

^c^RRMS: relapsing remitting multiple sclerosis.

^d^SPMS: secondary progressive multiple sclerosis.

^e^PPMS: primary progressive multiple sclerosis.

^f^PRMS: progressive relapsing multiple sclerosis.

Our method allowed us to identify the key themes from the small group discussions, such as accessibility, style, and exportable resources, and to compare these themes with the discussion summary statements that were most commonly voted as important by focus group participants (Table 2).

There were 2 main weaknesses in our focus group method. First, the small group facilitators reported some concern about fatigue. One facilitator suggested shortening the day by reducing the amount of time for questions at the end, whereas another suggested shortening the small group discussion periods from 15 to 10 min. Second, our selection of example videos was poor (ie, did not illustrate stylistic differences well), and we suggest that future projects choose videos more carefully to clearly show the differences in style. This might be accomplished by creating a few videos about the same subject in different styles, specifically for use in the focus group.

**Table 2:** The most important small group discussion summary statements (≥6 votes), as voted by focus group participants. The focus group participants were able to vote for the same statement multiple times.

Question		Votes, n	
**What topics/subjects would you like to see covered in an online course about MS^a^?**			
	Tips, management tools/activities to make living with MS easier, positive, and enhance quality of life		8
	What to expect for certain scenarios (ie, lesion location)		7
	Support and where to find it		7
	Guides for partners, children, public personnel, establishments, and health care providers (eg, general practitioner and allied health)		6
	Support services, events in local area		6
	Guides for types, symptoms		6
**What modes of delivery do you prefer?**			
	Video lecture (facing the audience)		12
	Keep it simple		11
	Link to other resources		6
**What would make an online course about MS useful to you?**			
	Interview people with MS		13
	Know more about MS		8
	Cater for a variety of learning styles		6
**What would make the course engaging for you?**			
	Plain English, simple language		14
	Consider different learning styles, for example, visual and reading—either options or a mix of presentation styles		10
	Speakers—engaging, warmth, humor, lived experience		9
	Speakers must be engaging. Academics in chairs speaking to each other=boring		7

^a^MS: multiple sclerosis.

### Development of Course Materials

In total, we created 53 videos, including 6 *MS perspectives videos* and 4 *community insights* videos, totaling 5 hours, 40 min, and 32 seconds of film. Only 4 used green screen animations. There were a total of 62 pages from the introduction through the completion of modules. On average, participants in the pilot study took approximately 2 hours to complete each module (Table 3), equating to 12 hours of total course content.

Our content development process had 4 main strengths. First, our core principles effectively shaped the tone of the course. We translated them into course-related materials in a variety of ways, including the following examples:

1. *Do no harm*. This influenced both the tone and the structure of the course. For example, while the content about MS symptoms, in general, was mandatory, videos detailing specific MS symptoms were made optional. This allowed participants to avoid exposure to information that might increase their symptom-related anxiety.
2. *Keep it positive*. In the feedback survey, instead of asking what was wrong with the course, we asked what could be improved.
3. *Share the journey*. Throughout the course, we choose to use the pronoun *we* instead of *you* to emphasize a sense of community. In addition, we individually greeted each participant who posted on the introduction discussion board in the first week of the open enrollments, thanking them for their participation and welcoming them to the course.

Second, having a single academic lead who oversaw all course content development was a very effective strategy, and we would recommend this approach to others. We found that it reduced duplication and contradiction and increased the continuity between sections. As all the written material (eg, text, quizzes, summaries, etc) had a single author, the course had a consistent style and pitch.

Third, we used a variety of different video styles. Not only is it more visually interesting but each video format also has particular advantages. The interview style is very flexible and allows for filming to be stopped between questions, which provides time for the academic lead and the presenter to workshop the upcoming question and response. Frequent pauses also mean that it is easy to reshoot the responses and only requires the presenter to remember their response to one question at a time. The conversational style allows for in-the-moment redirection of the presenter and follow-up questions by the academic lead. The conversational and interview styles require minimal upfront time investment by the presenters, which reduces the burden placed on them and may make them more likely to participate and enjoy participating. The direct-to-camera style allows for the concise delivery of information. However, it requires more upfront preparation, including memorization of a script. For this reason, this style of video was only presented by the academic lead.

Fourth, strong selection of video presenters from across the MS community (eg, people living with MS, caregivers, researchers, neurologists, etc). We found that the area experts made the best presenters, as they were comfortable speaking about their area of expertise. Inviting the presenters to workshop the script ahead of shooting increased their involvement and preparation and made them more comfortable when filming. We found that filming was improved by keeping things casual. This made presenters more comfortable, which both improved their performance and made them more likely to enjoy themselves.

The largest weakness of our content development was our ambitious timetable, which allowed approximately 10 months leading up to the first open enrollment. Given the amount of content needed, this rushed the process. In addition, because of financial and logistical constraints, the course materials were only made available in English. This limited the accessibility of the course.

**Table 3:** Demographics of the pilot study participants who completed the feedback surveys.

Demographics		Module 1 (n=51)	Module 2 (n=44)	Module 3 (n=41)	Module 4 (n=41)	Module 5 (n=36)	Module 6 (n=39)	Overall (n=29)
**Gender, n (%)**								
	Female	47 (92)	41 (93)	38 (93)	38 (93)	33 (92)	37 (95)	26 (90)
	Male	4 (8)	3 (7)	3 (7)	3 (7)	3 (8)	2 (5)	3 (10)
**Education, n (%)**								
	Secondary school	6 (12)	4 (9)	4 (10)	3 (7)	4 (11)	5 (13)	2 (7)
	Occupational certificate or diploma	7 (14)	7 (16)	5 (12)	8 (20)	5 (14)	7 (18)	7 (24)
	Undergraduate degree	16 (31)	14 (32)	14 (34)	13 (32)	10 (28)	12 (31)	8 (28)
	Postgraduate degree	22 (43)	19 (43)	18 (44)	17 (42)	17 (47)	15 (39)	12 (41)
**MS^a^community roles, n (%)**								
	Person with MS	32 (63)	29 (66)	25 (61)	27 (66)	24 (67)	26 (68)	17 (59)
	Family member or friend	12 (24)	10 (23)	10 (24)	9 (22)	10 (28)	8 (21)	7 (24)
	Caregiver	3 (6)	2 (5)	2 (5)	3 (7)	3 (8)	2 (5)	0 (0)
	Allied health professional	4 (8)	5 (11)	3 (7)	4 (10)	2 (6)	4 (10)	4 (14)
	MS nurse	2 (4)	2 (5)	2 (5)	2 (5)	2 (6)	2 (5)	2 (7)
	General practitioner	0 (0)	0 (0)	0 (0)	0 (0)	0 (0)	0 (0)	0 (0)
	Neurologist	1 (2)	1 (2)	0 (0)	1 (2)	1 (3)	0 (0)	0 (0)
	Advocate	1 (2)	1 (2)	0 (0)	1 (2)	1 (3)	1 (3)	0 (0)
	Service provider	2 (4)	2 (5)	3 (7)	2 (5)	1 (3)	1 (3)	2 (7)
	Researcher	4 (8)	2 (5)	2 (5)	1 (2)	0 (0)	0 (0)	0 (0)
	Other	6 (12)	4 (9)	4 (10)	4 (10)	6 (17)	4 (10)	5 (17)
**MS onset phenotype, n (%)**								
	Primary progressive	1 (3)	2 (5)	1 (2)	2 (5)	2 (6)	2 (5)	1 (3)
	Progressive relapsing	1 (3)	0 (0)	0 (0)	0 (0)	0 (0)	0 (0)	0 (0)
	Relapsing remitting	18 (56)	16 (36)	15 (37)	15 (37)	12 (33)	14 (36)	11 (38)
	Secondary progressive	9 (28)	8 (18)	8 (20)	8 (20)	8 (22)	8 (21)	3 (10)
	I am not sure	3 (9)	3 (7)	1 (2)	2 (5)	2 (6)	2 (5)	2 (7)
Age (years), mean (SD)		52.1 (12.3)	51.9 (11.4)	52.8 (12.4)	53.2 (12.4)	55.3 (12.2)	52.8 (12.3)	51.9 (12.8)
MS disease duration, mean (SD)		10.7 (8.7)	11.7 (9.2)	11.4 (8.4)	10.2 (8.2)	11.5 (8.1)	11.0 (8.4)	7.8 (5.8)
Time to completion (hours), mean (SD)		1.9 (1.0)	2.1 (0.9)	1.9 (0.9)	1.6 (0.9)	2.1 (1.1)	1.7 (1.0)	2.2 (1.2)

^a^MS: multiple sclerosis.

### Pilot Study

A total of 97 people enrolled in the course of the pilot study. Of those, 51 provided feedback on at least one section of the course. Across all surveys, a minimum of 90% (26/29) of the respondents were female, and a minimum of 59% (17/29) were people living with MS. The pilot study participant demographics are presented in Table 3.

The pilot study had 2 main strengths. First, the group was reasonably representative of the MS community, with a variety of MS community roles represented and a range of MS phenotypes. Second, we were able to pinpoint areas for improvement because we surveyed participants about each module and the overall course, which provided fine-grained data (shown in Multimedia Appendix 7). If we had simply asked about overall satisfaction, our ability to target underperforming areas would have been lost (compare part A of Multimedia Appendix 7 with part B of Multimedia Appendix 7).

The main weakness of the pilot study was the relative paucity of data. This was a fairly small study, and we did not make providing feedback a mandatory part of participating in the pilot. Therefore, we did not collect as much data as we could have. More data may have provided a more detailed or nuanced view of the course materials.

## Discussion

### Principal Findings

Overall, our process was effective and efficient. This resulted in the production of a successful health and medicine MOOC aimed at the MS community and interested laypeople with high participant satisfaction, retention, and recruitment and with no major technical errors. We believe that this project was a good value for money and that the funds were well spent. We estimate that without extensive in-kind support, the project costs would have exceeded AUD $500,000. The generosity of our project partners was essential to its success and to illustrate the community support for this work. We have identified 5 key lessons learned from our experience.

#### Community Support is Essential

The support that this project received from the community, UTAS, and Multiple Sclerosis Limited was critical. Without it, this project would not have been possible. UTAS’ institutional support was particularly extensive. We were able to leverage the expertise, experience, and resources (particularly the custom online course platform) of the WDREC to achieve high cost-effectiveness. This was a sizable advantage; without it, the project would have been significantly more expensive, challenging, and time-consuming. Community buy-in and support also performed several other essential functions for the project. This allowed us to integrate the community from the outset. This led to collaboration, co-design, and engagement and allowed us to identify, understand, and tailor course content for our audience early on.

#### Stakeholder Involvement Improves Content Quality

There are many reasons to engage with stakeholders, including social justice and utilitarian motivations [17]. From a social justice perspective, there are compelling ethical reasons to involve stakeholders in the process early and often. From a utilitarian perspective, we found that early stakeholder involvement allowed us to identify the preferences of our target audience at the beginning of the process and helped to ensure that the content we created was appropriate. For example, we were surprised by the importance placed by focus group participants on the tone of the course. They indicated that they wanted a warm, good-natured tone. We incorporated this into the course content. This was a significant benefit to our project.

#### Plan for Research From the Beginning

We found that the best time to integrate research into an MOOC is at the beginning, while design and development are underway. This makes it possible to incorporate research tools directly into the course. It also allows sufficient time to produce tools that may be missing and require development. Early preparation also allowed the pilot study to function as a test of the course evaluation survey, which is now available for all course completers to provide feedback.

#### The Academic Lead and Project Management Team Ensure a Consistent Voice

A dedicated academic lead and project management team ensured that there was a consistent voice and style throughout the course content, accelerated course development, and minimized discrepancies in the course material. As this was their primary project throughout the year, they ensured that our ambitious timeline was met. They worked together to establish the textual, visual, and tonal styles of the course.

#### A Network of Collaborators Is a Key Resource

Our network of collaborators provided area experts from across the MS community, provided input on the course material, and, in the case of the WDREC, allowed us the immense benefit of their experience. Their generosity with their time made this project possible within the time and budget constraints presented above. Furthermore, collaborating at every level of this project has ensured that the community is involved and that stakeholders are represented, including in the authorship of this manuscript.

### Strengths and Limitations

The main strength of this research was the diverse group of MS community members involved in all aspects of course development. The main limitations of this work were that it primarily involved Australian participants and was limited to those fluent in English. The web-based components further required the ability to access and use digital platforms.

### Conclusions

The process behind the development of the Understanding MS MOOC was extensive, but each component contributed to a successful outcome. However, although this process was successful for us, it is important to recognize that this is a single project. We hope that by providing our material here, we will encourage others to use them in future work, allowing for meaningful comparisons in course development methodology and outcomes.

## Appendix

Multimedia Appendix 1 Initial course syllabus. PwMS: person living with Multiple Sclerosis.

Multimedia Appendix 2 Focus group small group discussion questions.

Multimedia Appendix 3 Finalized course map for the first open enrolment.

Multimedia Appendix 4 Example quiz. Correct answers are bolded.

Multimedia Appendix 5 List of discussion prompts and activities from the first iteration of the Understanding Multiple Sclerosis online course.

Multimedia Appendix 6 An example pilot study survey querying participant feedback on the overall course.

Multimedia Appendix 7 Pilot study results showing the percentage of participants who reported being satisfied or very satisfied with (A) the course sections (modules) and the course overall, and (B) each component (eg, video and text) of each course section and the course overall.

## Abbreviations

Menzies: Menzies Institute for Medical Research
MOOC: massive open online course
MS: multiple sclerosis
UTAS: University of Tasmania
WDREC: Wicking Center for Dementia Research and Education
