# Supplementary material for: Building a Successful Massive Open Online Course About Multiple Sclerosis: A Process Description
Source: J Med Internet Res. 2020 Jul 29;22(7):e16687. doi: 10.2196/16687 (PMC7424472; doi:10.2196/16687)
Supplement: Multimedia Appendix 1 [file jmir_v22i7e16687_app1.docx]

**Appendix 1:** Initial course syllabus. PwMS: person living with MS

| **Module** | **Content** |
| --- | --- |
| **Week 0:**  **Introduction to the Understanding MS MOOC** | - 1. **Welcome participants**   Short welcome message stating the aim of the MOOC and our hope that it will be of use to the MS community. |
|  | - 1. **What is the MOOC about? Who is the MOOC for?**   Restate the aim of the MOOC and describe the intended audience, including all members of the MS community and those interested in learning more about MS. |
|  | - 1. **What will participants learn?**   Printable syllabus and compilation video (video comprised of clips from the videos that will appear throughout the course in a sort of course preview.) |
|  | **0.4 Team biographies i.e. pictures and brief bios about each team member/expert** |
| **Week 1: Module 1 Brain science and MS pathology** | - 1. **Introduction to brain science** - What is the brain? - What is its function? |
|  | - 1. **CNS structure and function** - Concisely discuss the components of the CNS (brain and spinal cord), including neurons and myelin sheath. - Discuss their function in the body, including their impact on all other body systems. - Explain that the structure and function of the CNS underlies MS symptoms. |
|  | - 1. **MS pathology** - Discuss the three main components of MS pathology: inflammation, neurodegeneration, the breakdown of CNS tolerance and repair mechanisms. - Highlight the anatomy behind the pathology, illustrating the effects of MS by showing which parts of the CNS are involved. - Mention the involvement of the immune system via inflammation. |
|  | **QUIZ** |
| **Week 2: Module 2 MS overview** | **2.1 MS demographics**   - Show the demographics of MS as a series of images using basic people symbols with people with MS highlighted in a bright color. Show the basic prevalence (1 out of X people), then show how that relates to a city (i.e. X people in Sydney), a country (X in Australia), and then globally (X in the world). - Show the ratio of women to men, and illustrate the latitudinal gradient. |
|  | **2.2 MS symptoms**   - Discuss average age of onset, and common symptoms of MS, such as optical neuritis, numbness, etc. - Emphasise the variability in symptoms between people, reiterating the underlying pathology. |
|  | **2.3 Common issues and challenges related to MS**   - Discuss issues and challenges that demand changes in personal routine. - In particular, address challenges related to mobility issues, from walking to self-care. - Include methods used to address challenges. |
|  | **2.4 Disease course**   - Discuss different courses (PPMS, RRMS, and SPMS) - Illustrate a typical course of each type - Emphasise the variability in MS progression - End with question of how you minimise the chances of a more aggressive course. |
|  | **QUIZ** |
| **Week 3: Module 3 Epidemiology and concept of risk** | **3.1 Understanding the concept of risk**   - risk ratios, - population vs personal risk, - what does ‘double’ or ‘half’ the risk actually mean? |
|  | **3.2 Assessing individual risk**   - How do we determine an individual’s risk of MS – risk assessment tools - How accurately can we determine individual risk? - What population risk evidence means for the individual |
|  | **3.3 Introduction to epidemiology**   - Studying populations - Importance of evidence/levels of evidence - Role of epidemiology in public health (include historical example of how epidemiology used to explain disease) - Epidemiological study design (brief and in lay terms) – retrospective case-control, prospective cohort, meta-analyses and systematic reviews |
|  | **QUIZ** |
| **Week 4: Module 4**  **MS risk factors** | **4.1 Major non-modifiable risk factors**   - Age - Gender (reiterate demographic knowledge) - Genetic risk factors – interaction between genetic and lifestyle risk factors - Exposure to pathogens (e.g. Epstein Barr) |
|  | - 1. **Major modifiable risk factors** - Vitamin D level - Sunlight exposure - Smoking - BMI |
|  | **4.3 Myths and controversies** – what are sometimes perceived to be, but are not risk factors for MS |
|  | **QUIZ** |
| **Week 5: Module 5**  **Treatments** | **5.1 Treatment types**   - Modifiable risk factor interventions - Symptom management - DMT |
|  | **5.2 Modifiable risk factor interventions**   - What works? - What is unproven? - Myths/what doesn’t work? - A healthy and active lifestyle |
|  | **5.3 Symptom management**   - Corticosteriods - Pain management - Mobility assistance |
|  | **5.4 DMT**   - Types of DMT - Uses for DMT (largely limited to RRMS) - Benefits of DMT - Side effects of DMT - Knowledge gaps about DMT |
|  | **QUIZ** |
| **Week 6: Module 6**  **Living with MS** | **6.1 Aside from the treatment of MS, there are several other aspects to living with MS, including:**   - Economics - Employment - Communication - Sources of support |
|  | **6.2 Economics of MS**   - Direct costs of MS (medication, treatment, etc.) - Indirect costs of MS (absenteeism, presenteeism, changing or leaving work, costs to family and friends who assist with MS-related issues, house renovation, etc.) |
|  | **6.3 Employment**   - Employment may be impacted by fatigue, mobility issues or other MS-related issues - Absenteeism, presenteeism - Change careers - Cut back on hours - Leaving paid employment |
|  | **6.4 Communication**   - Between PwMS and employer - Between PwMS and GP - Between PwMS and allied health - Between PwMS and specialist - Between PwMS and carer |
|  | **6.5 Sources of support**   - Family & friends - Professional carers - Allied health providers (therapists, physios, etc.) - GP - MS Societies - Neurologist |
|  | **6.6 Course Summary** |
|  | **QUIZ** |
