# Supplementary material for: Building a Successful Massive Open Online Course About Multiple Sclerosis: A Process Description
Source: J Med Internet Res. 2020 Jul 29;22(7):e16687. doi: 10.2196/16687 (PMC7424472; doi:10.2196/16687)
Supplement: Multimedia Appendix 2 [file jmir_v22i7e16687_app2.docx]

**Appendix 2:** Focus group small group discussion questions

*Question 1*: What subjects/topics would you like to see covered in an online course about MS?

Example subjects:

- Brain science
- Symptoms
- Risk factors
- Treatments
- Living with MS
- Working with MS
- Communicating about MS

*Question 2:* What modes of delivery to you prefer?

Example modes of delivery:

- Video (one person delivering a lecture to the camera)
- Video (two people in conversation)
- Text
- Animation
- Photos
- Graphs
- Discussion board
- Quizzes

*Question 3:* What would you like to get out of an online course about MS? What would make an online course about MS useful to you?

Please complete the following sentence “This course would be useful to me if ….”

Examples:

- If I felt like I had increased my knowledge about MS and this helped me in practical ways.
- If there was a sense of community with fellow participants.

*Question 4:* If people do not engage with the course and its content it will not be useful. What would make the course engaging for you?

Examples:

- If I found the language used was easily understood
- If I found the topics of relevance to me and my condition at this time
