# Supplementary figures and images for: Building a Successful Massive Open Online Course About Multiple Sclerosis: A Process Description
Source: J Med Internet Res. 2020 Jul 29;22(7):e16687. doi: 10.2196/16687 (PMC7424472; doi:10.2196/16687)

**Appendix 3:** Finalized course map for the first open enrolment


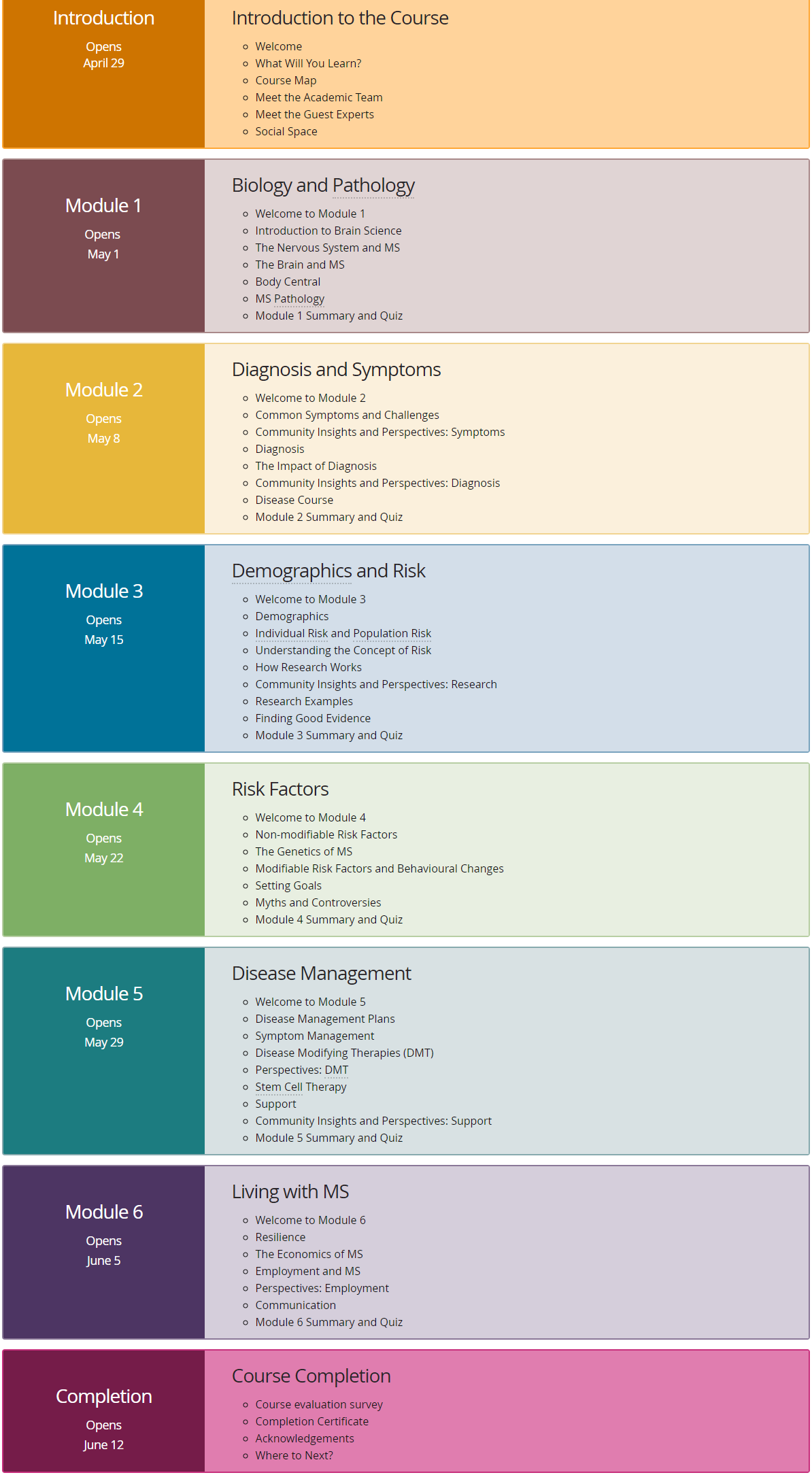

Supplement: Multimedia Appendix 3 [file jmir_v22i7e16687_app3.docx]

**Appendix 6:** Example pilot study survey querying participant feedback on the course overall.


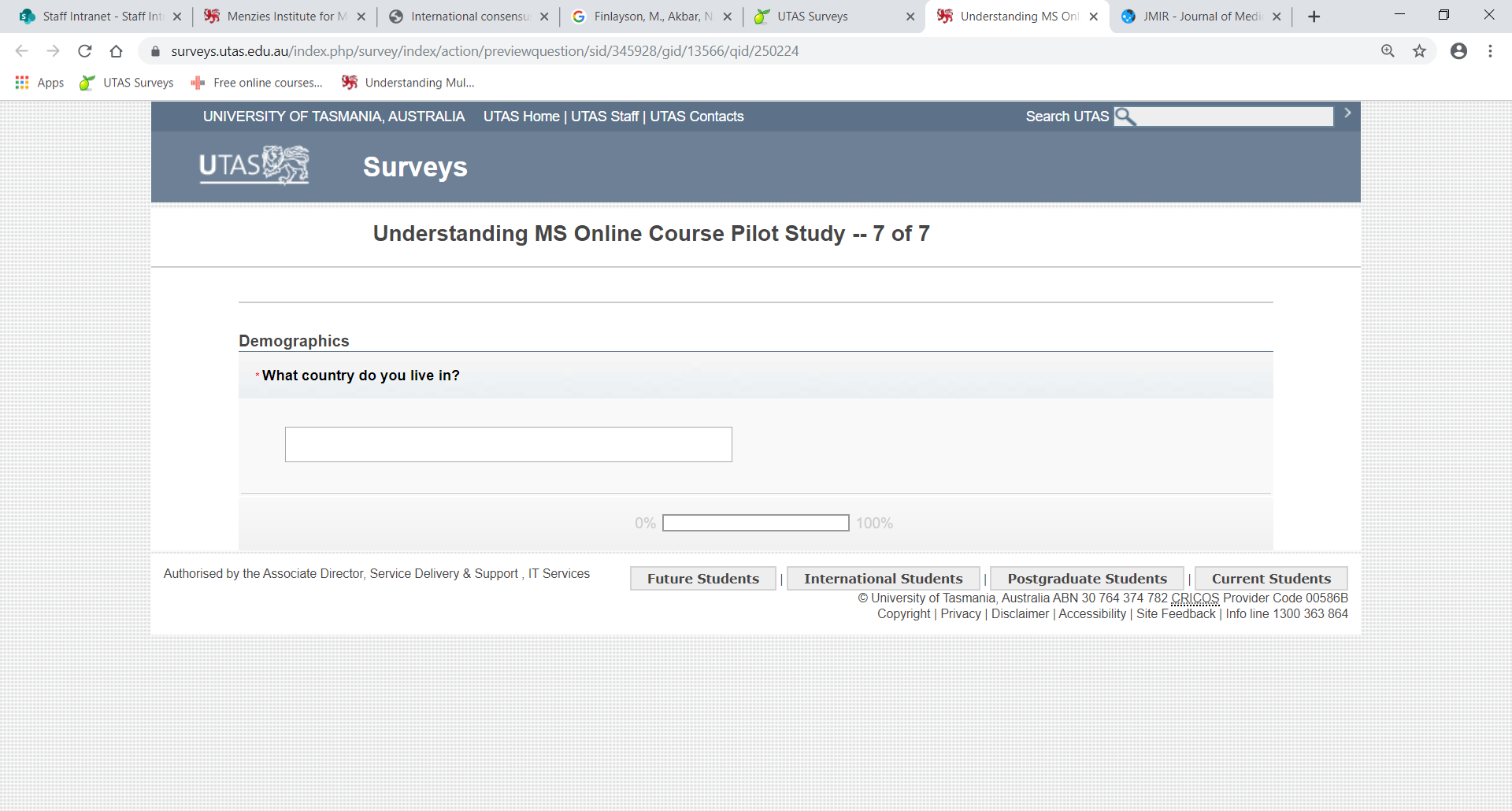


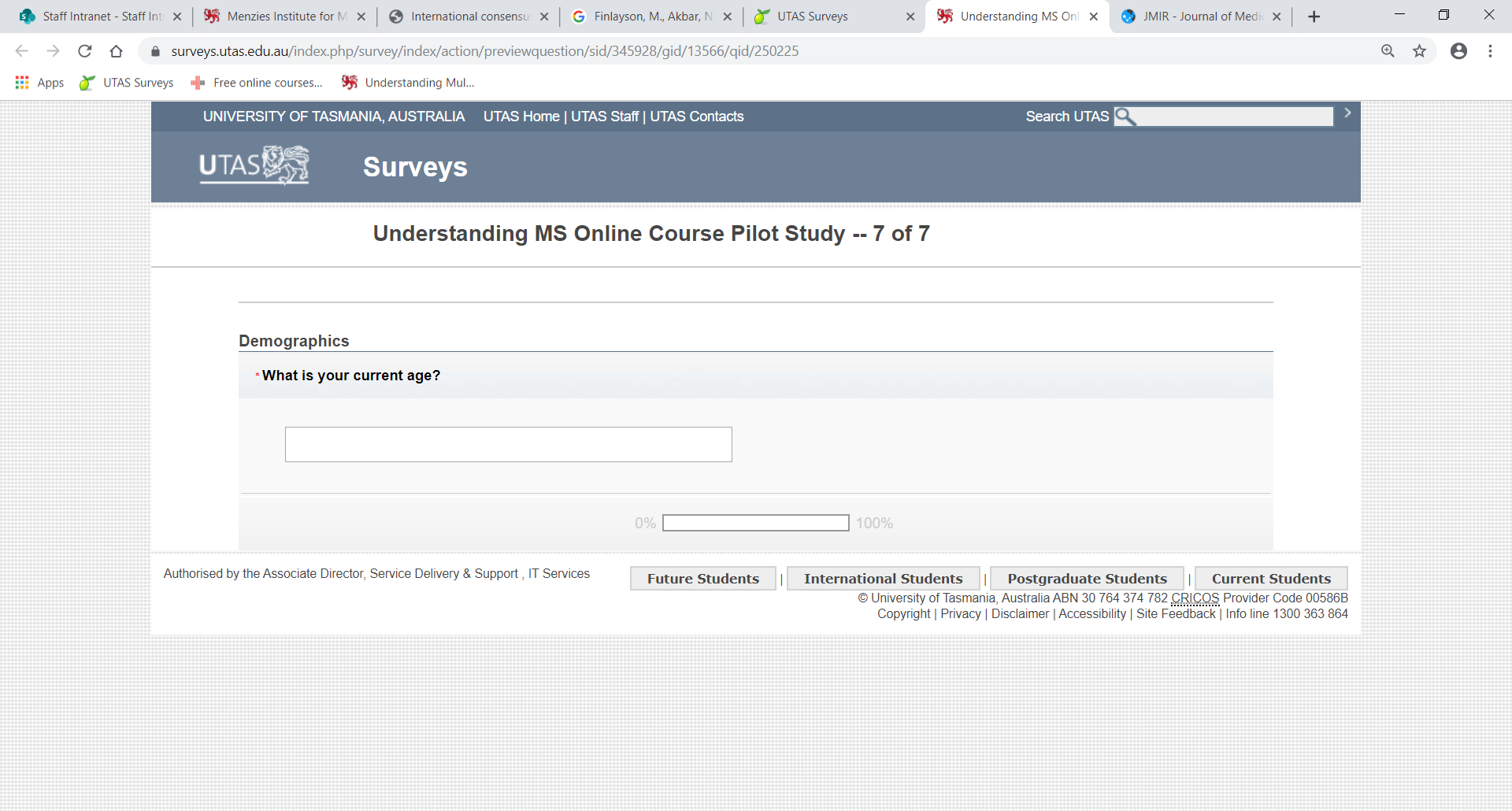


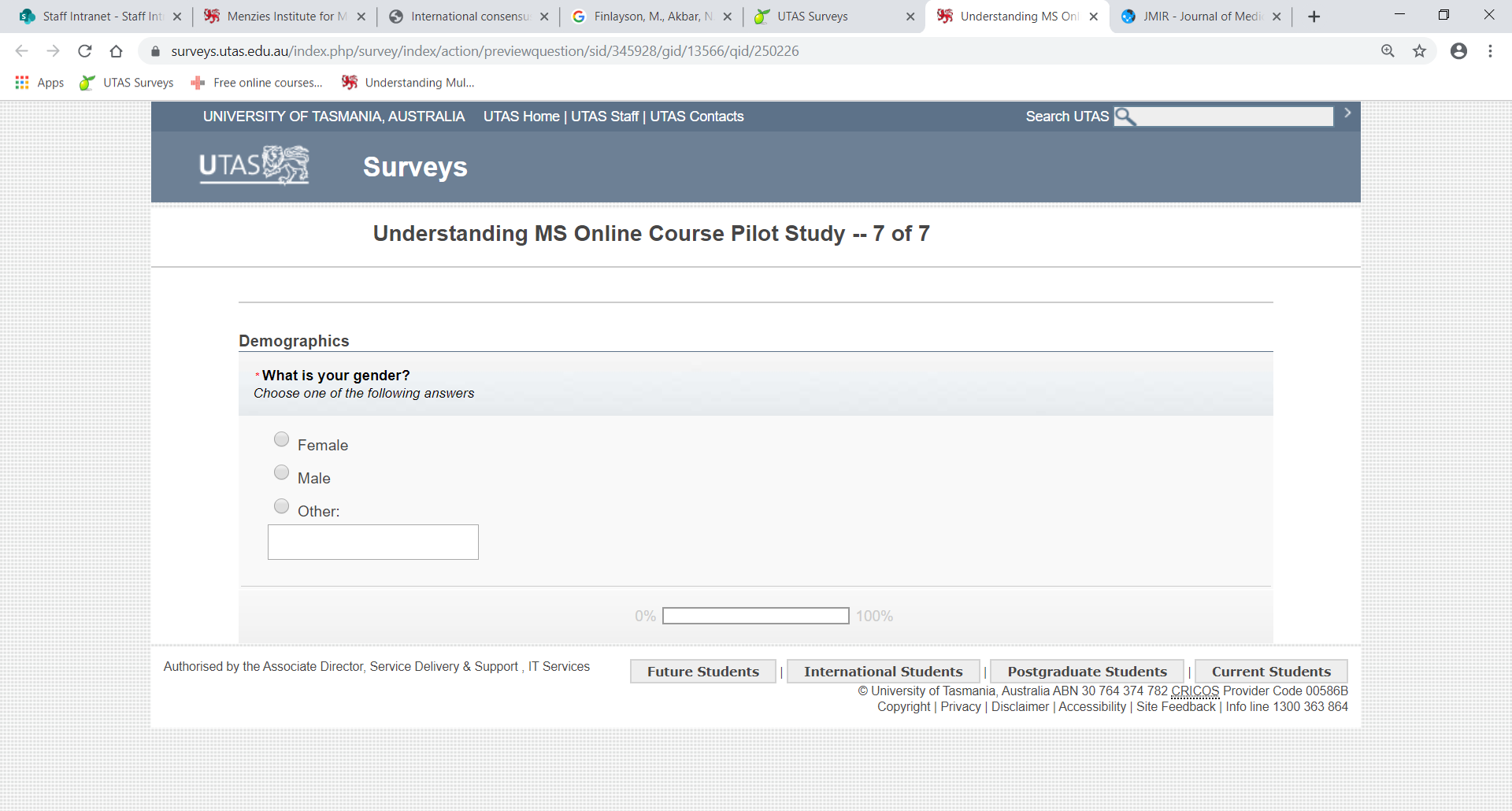


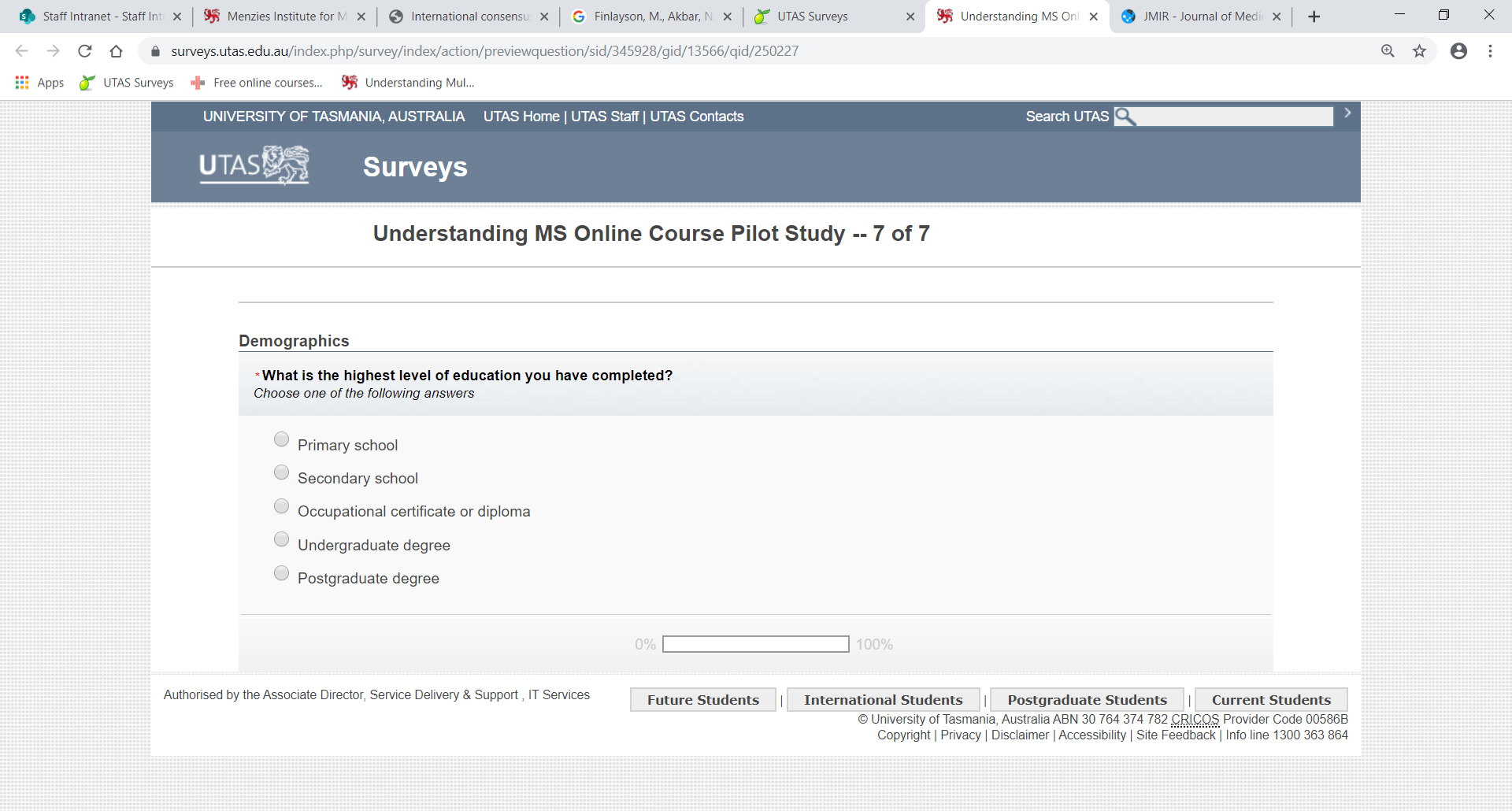


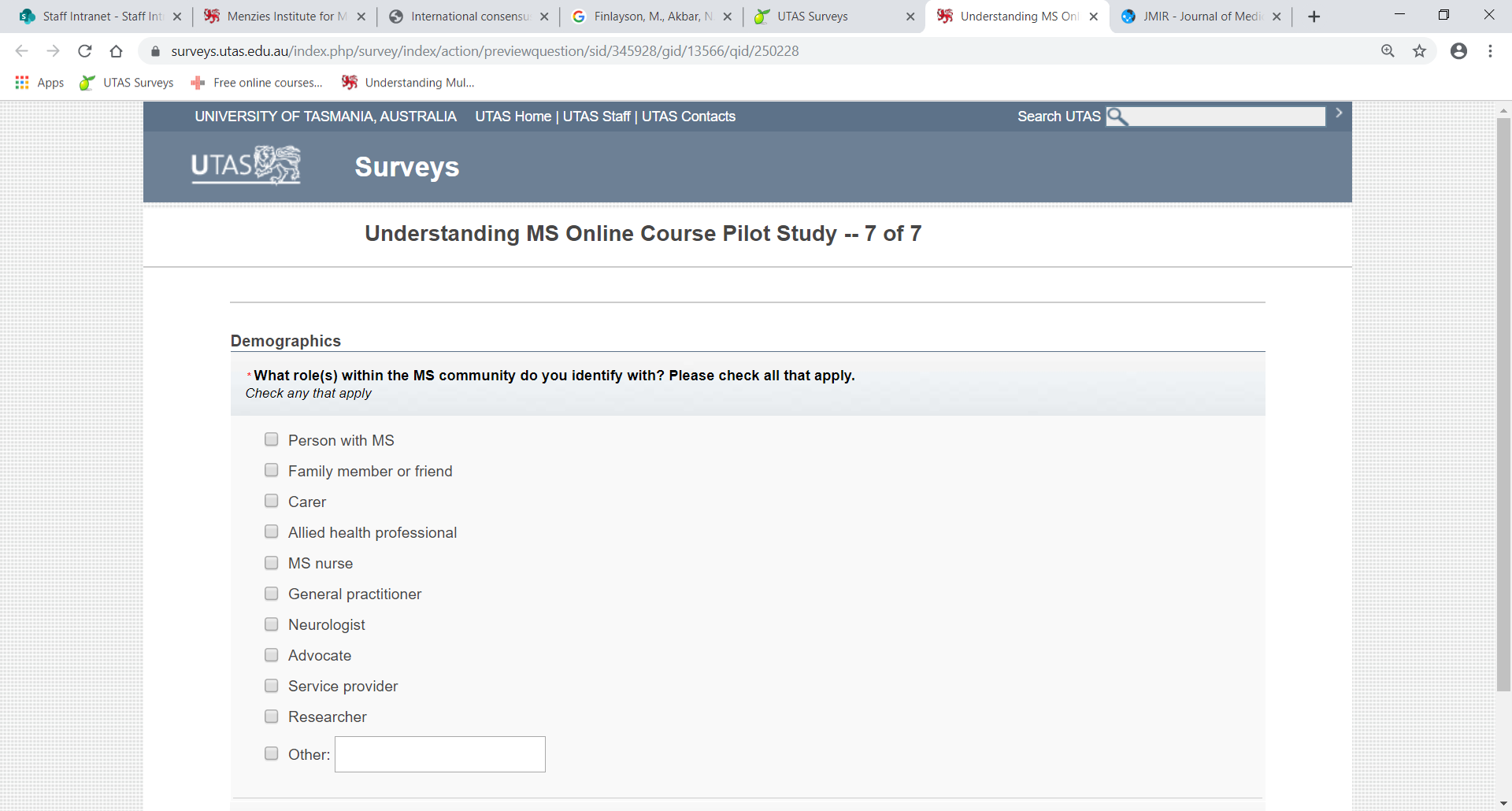


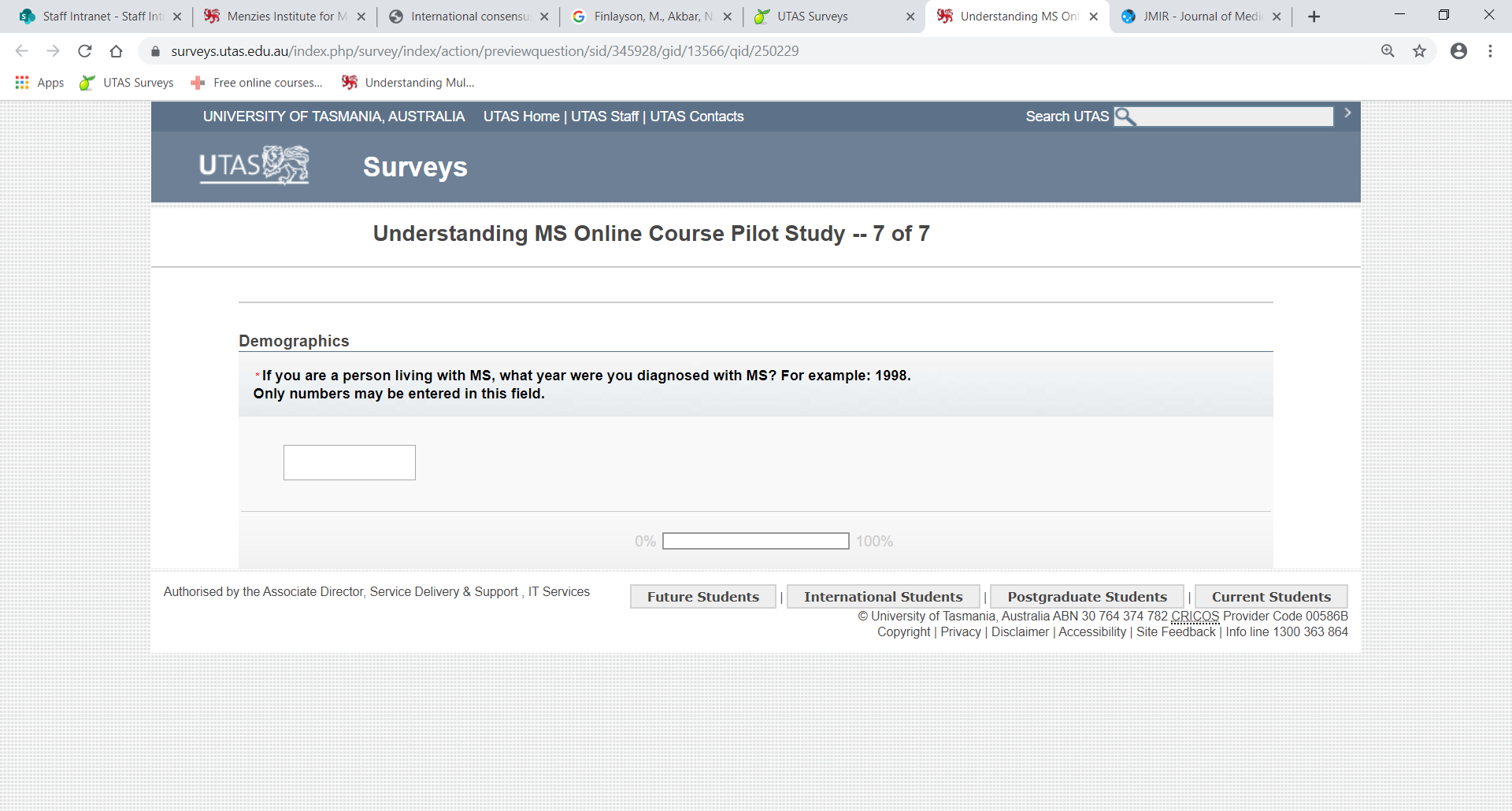


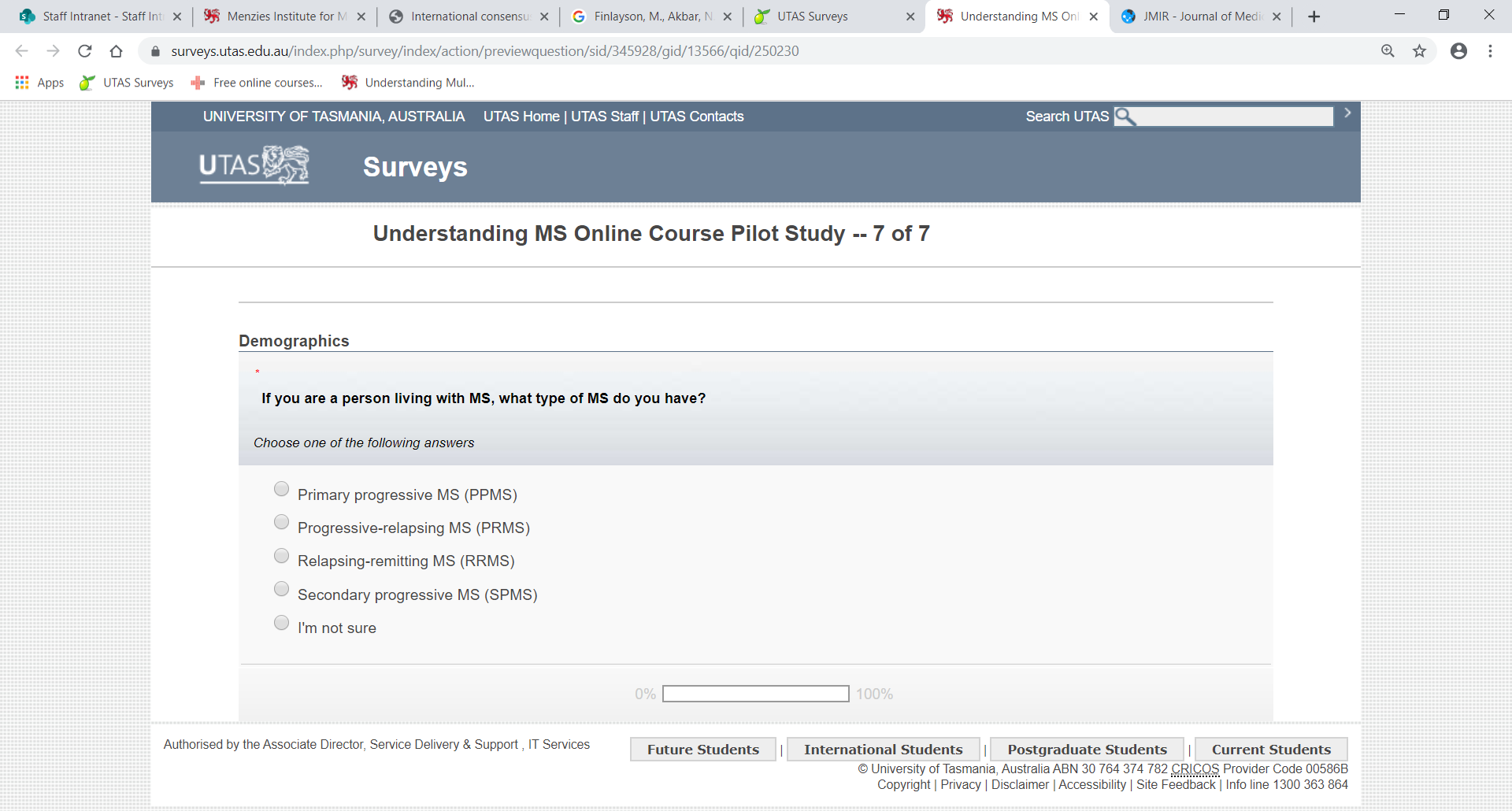


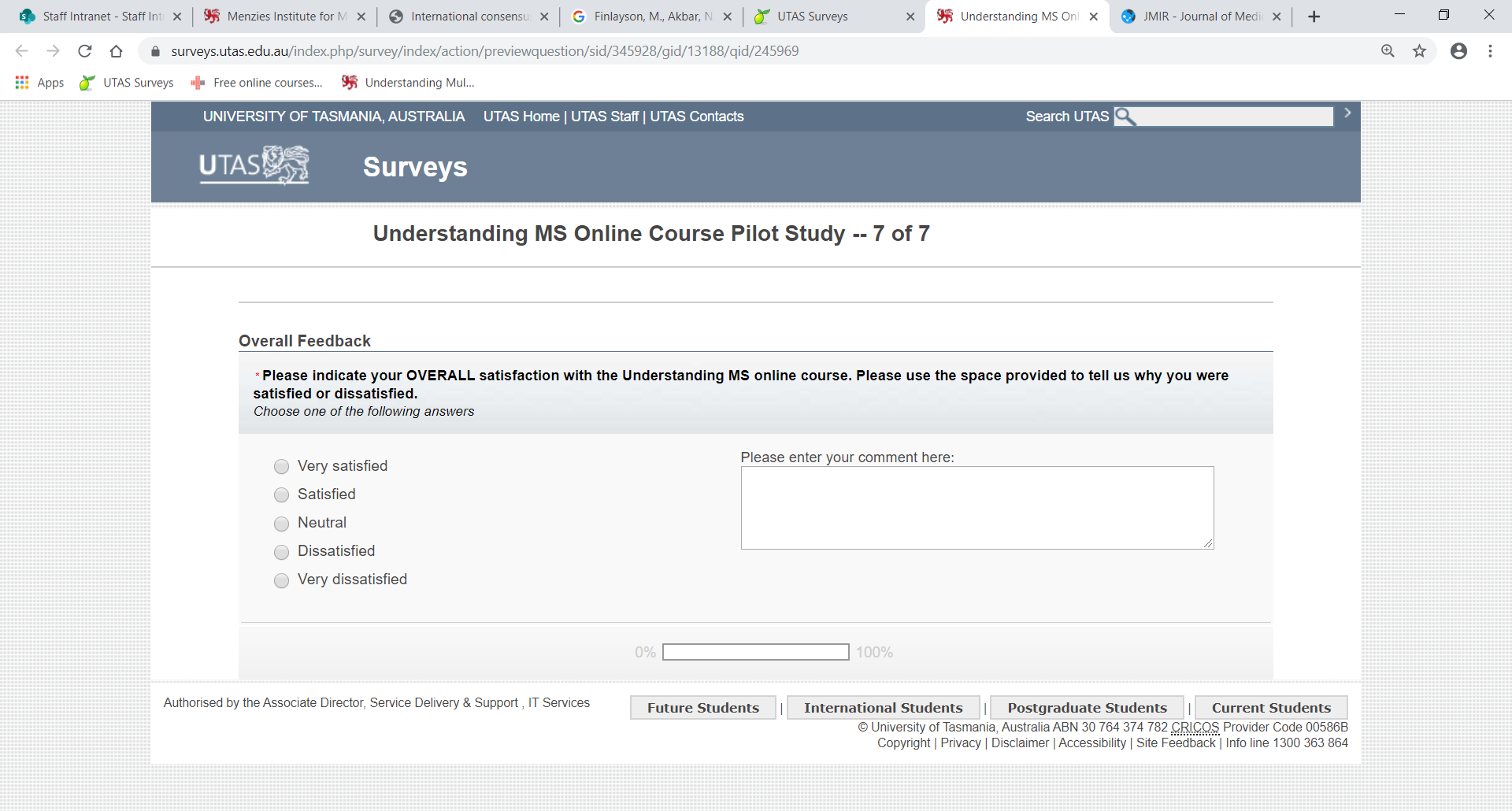


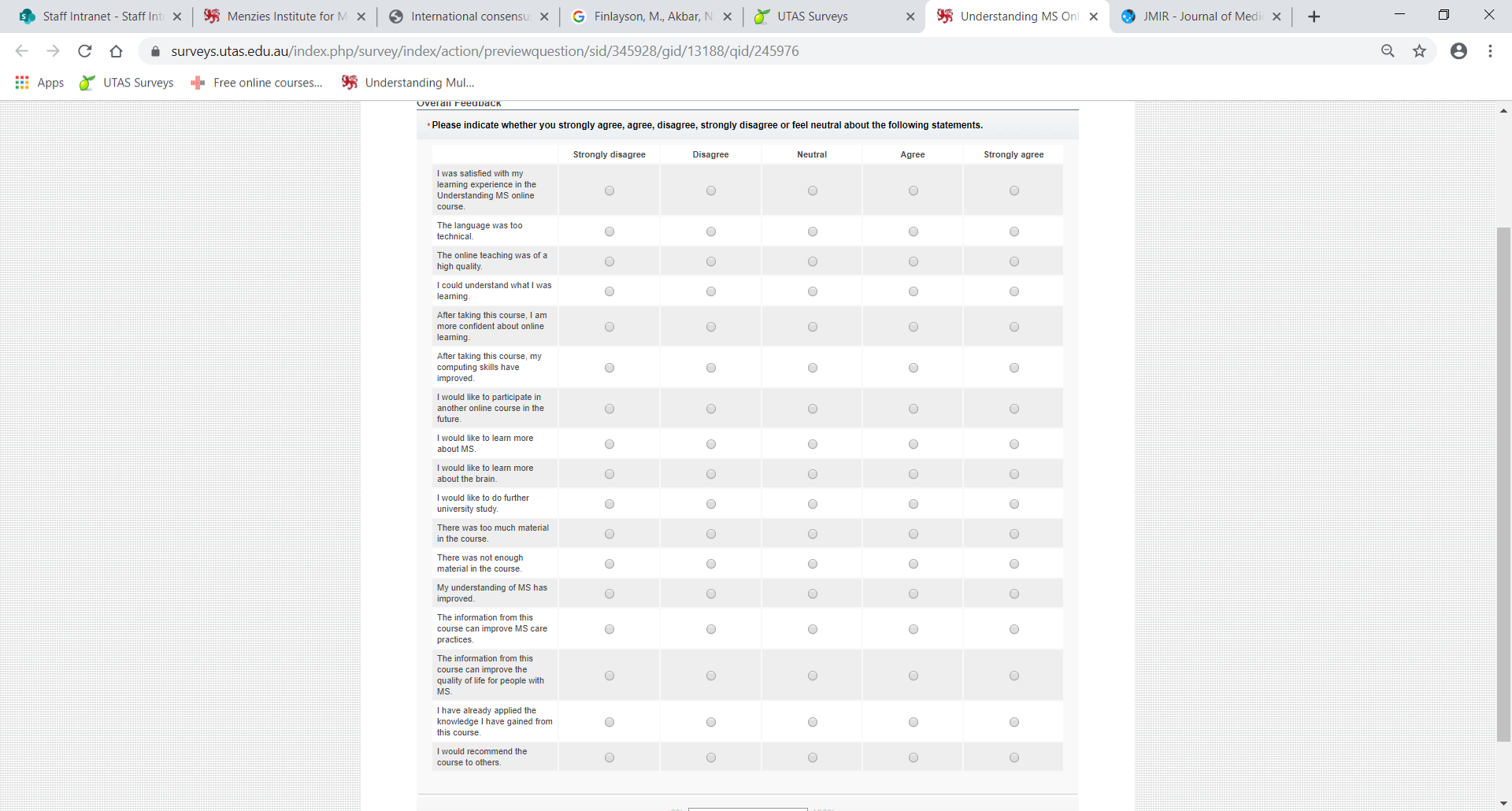


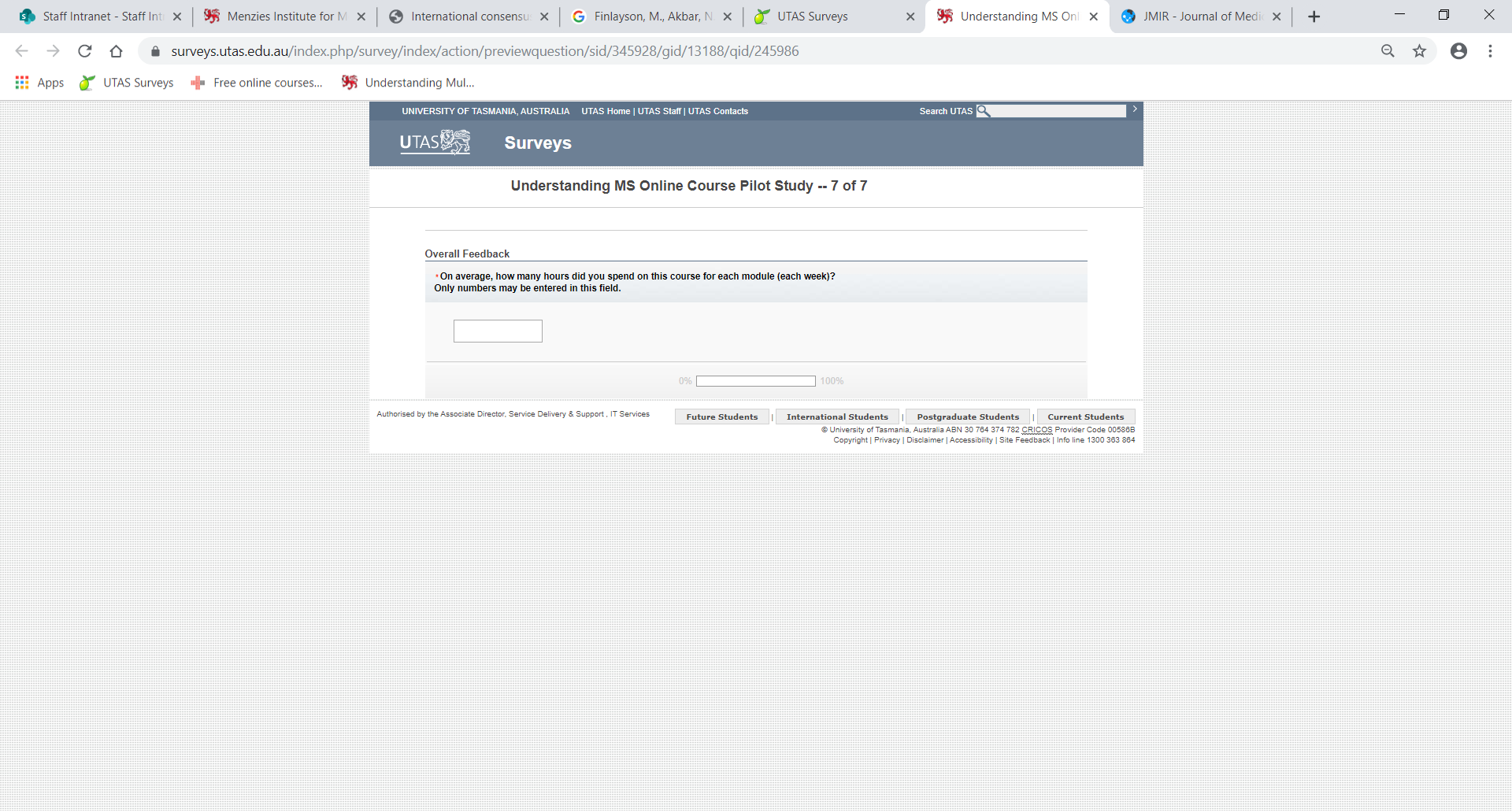


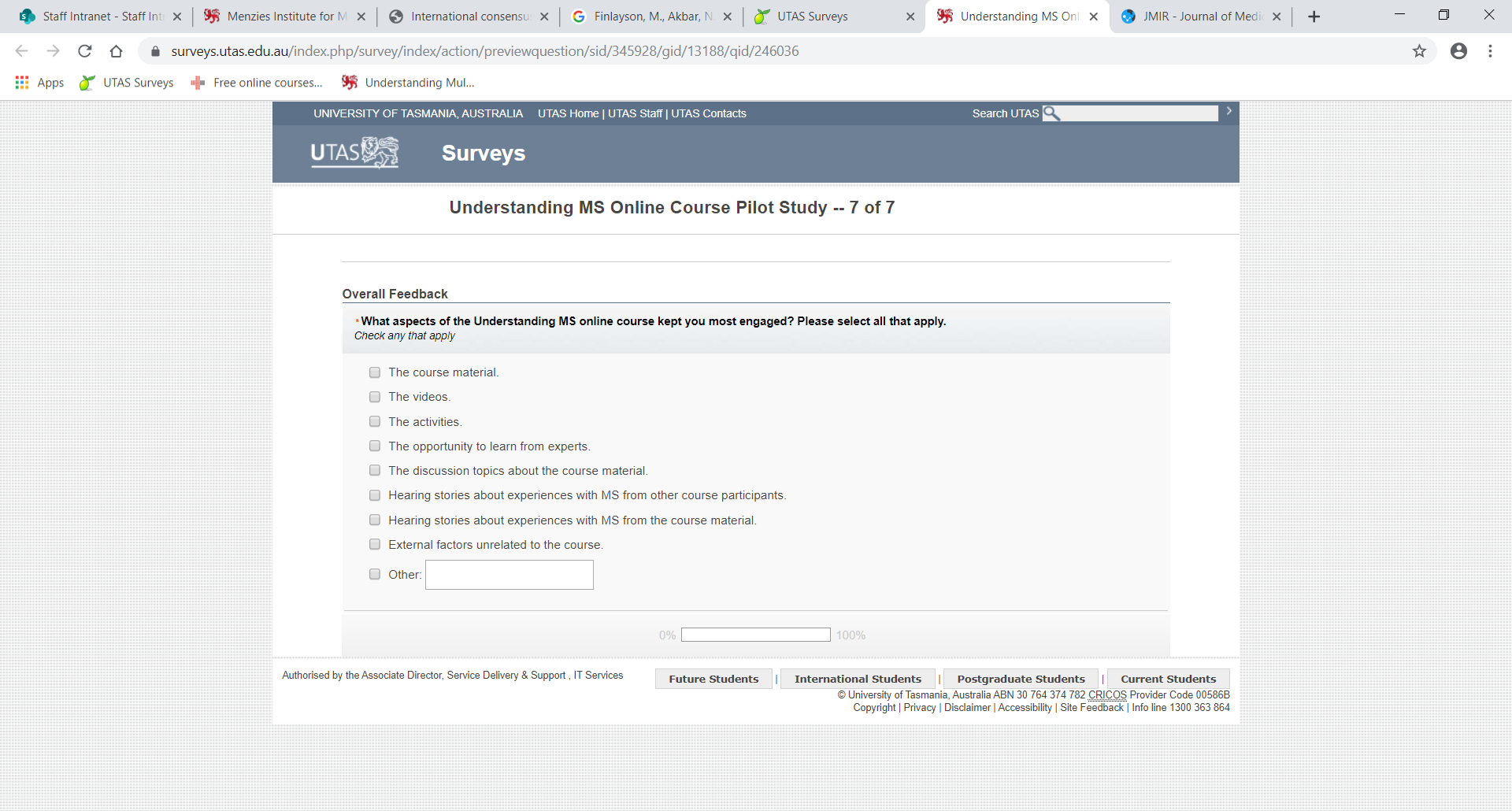


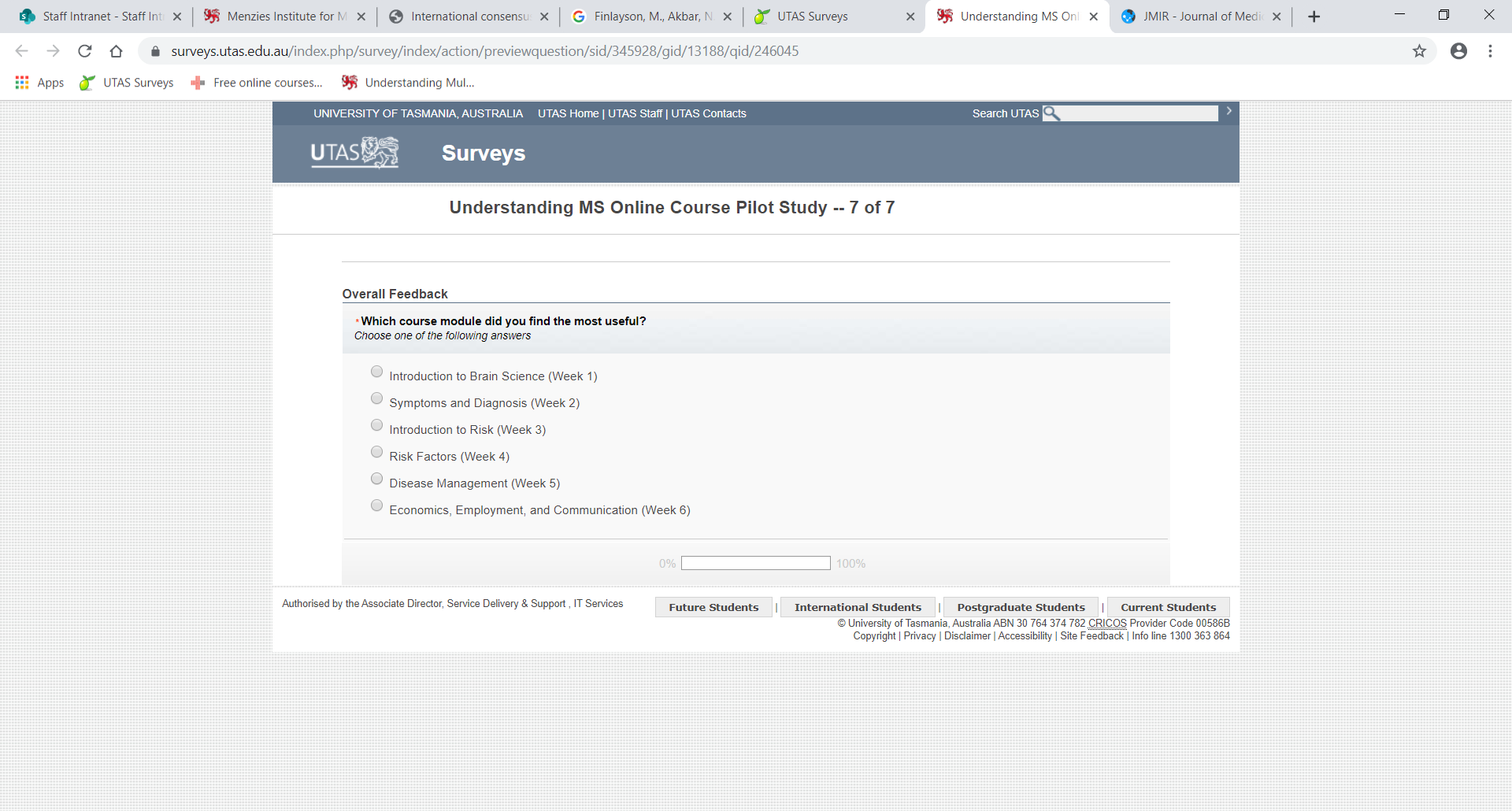


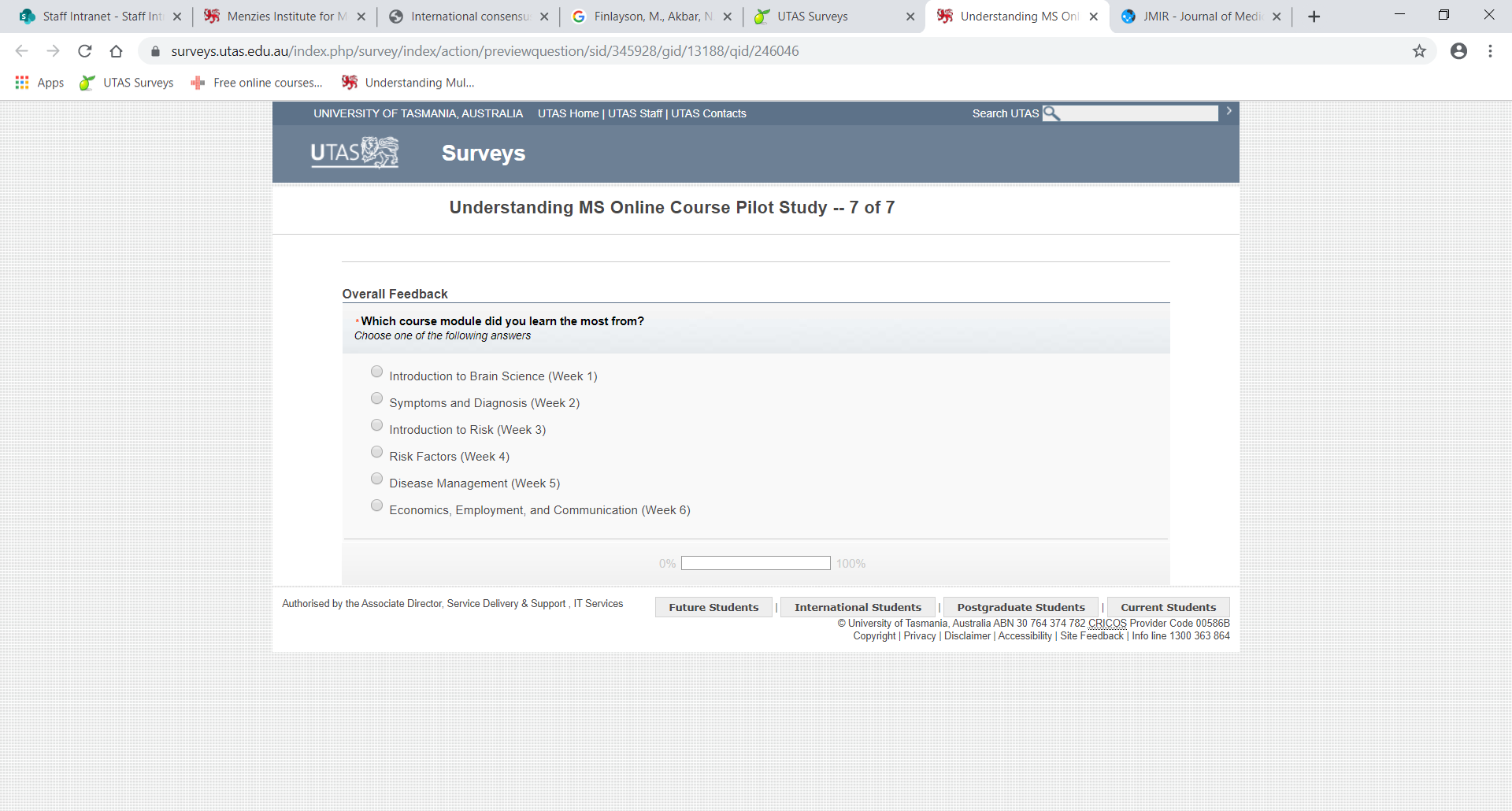


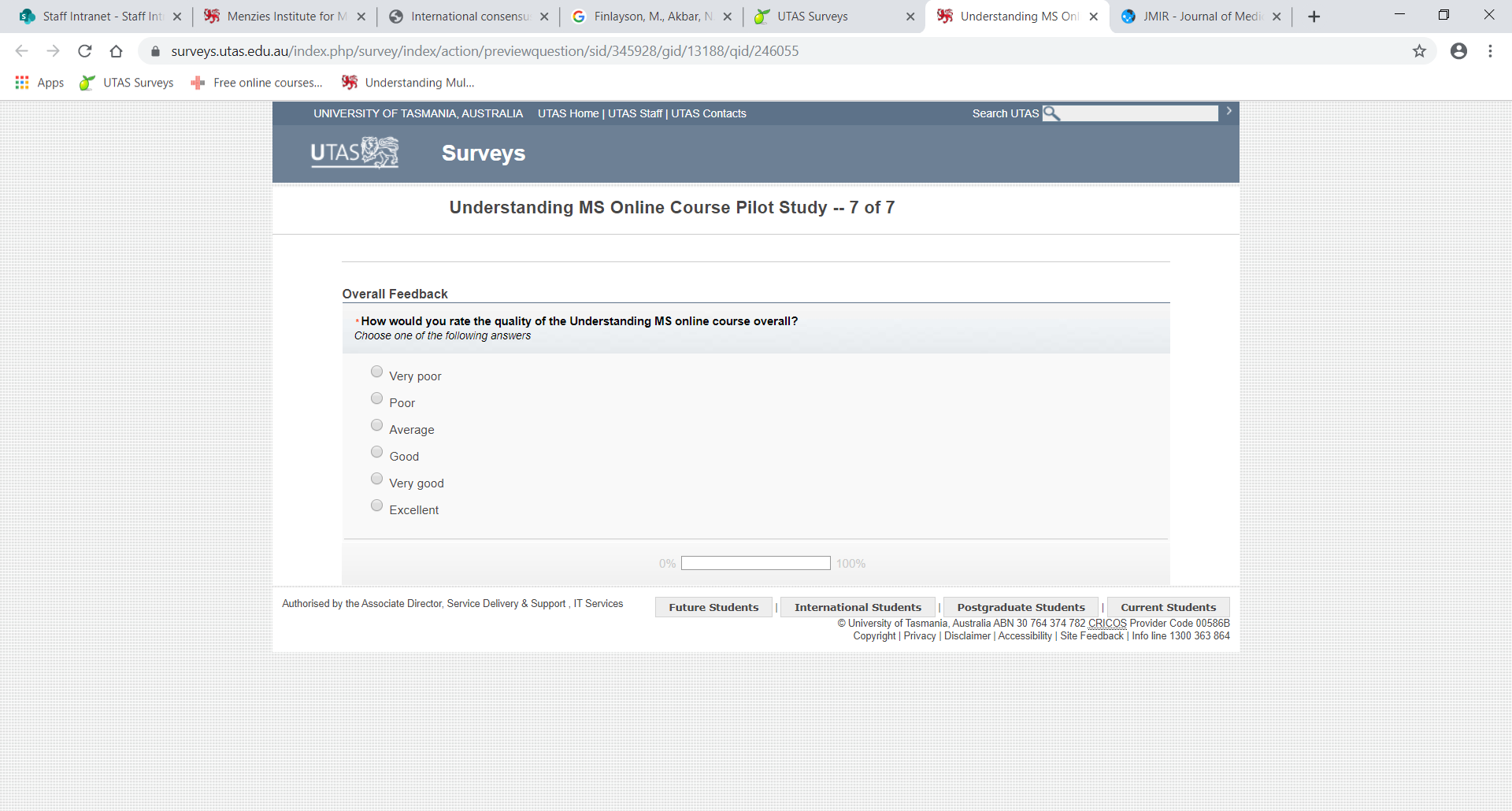


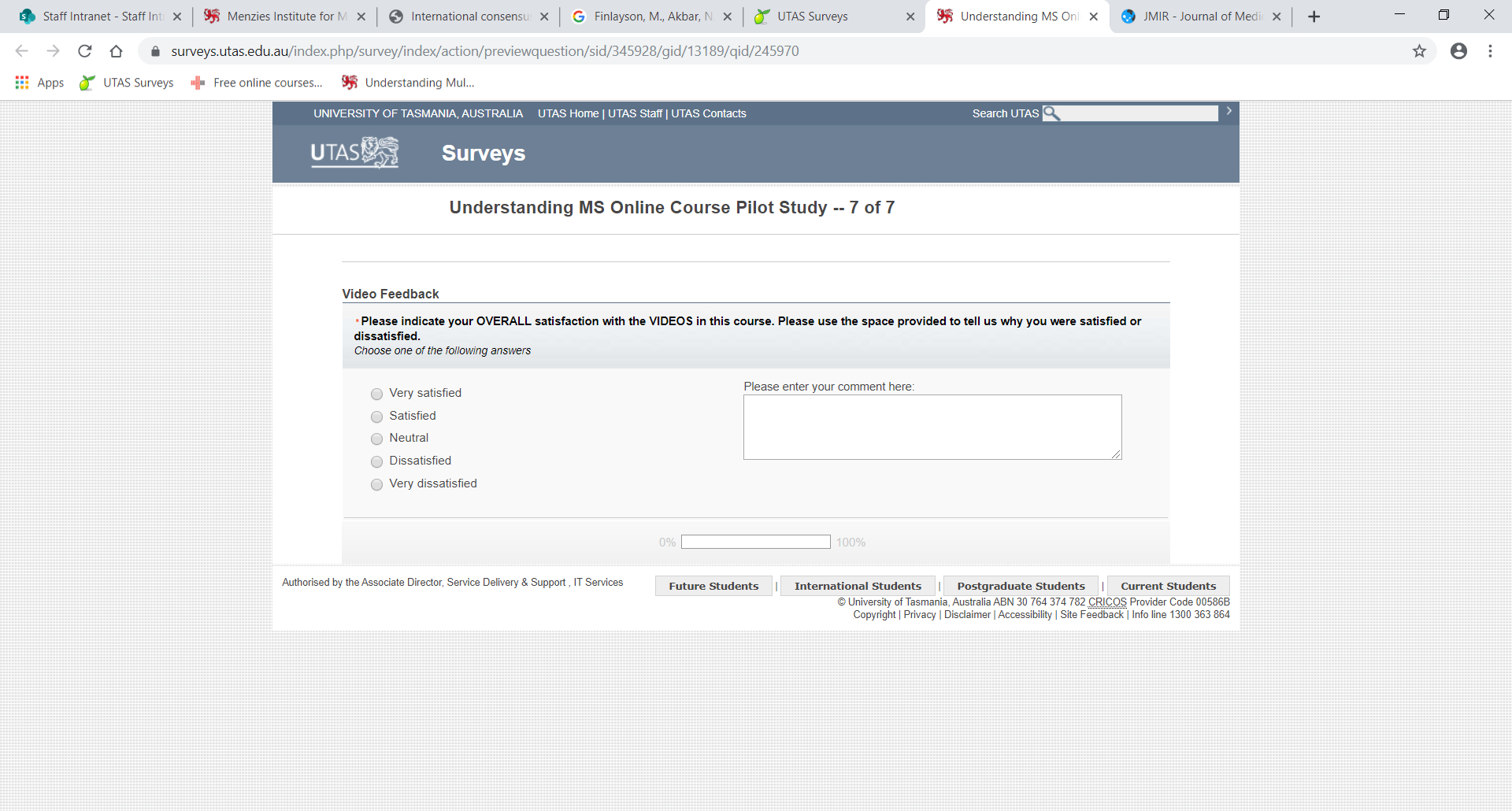


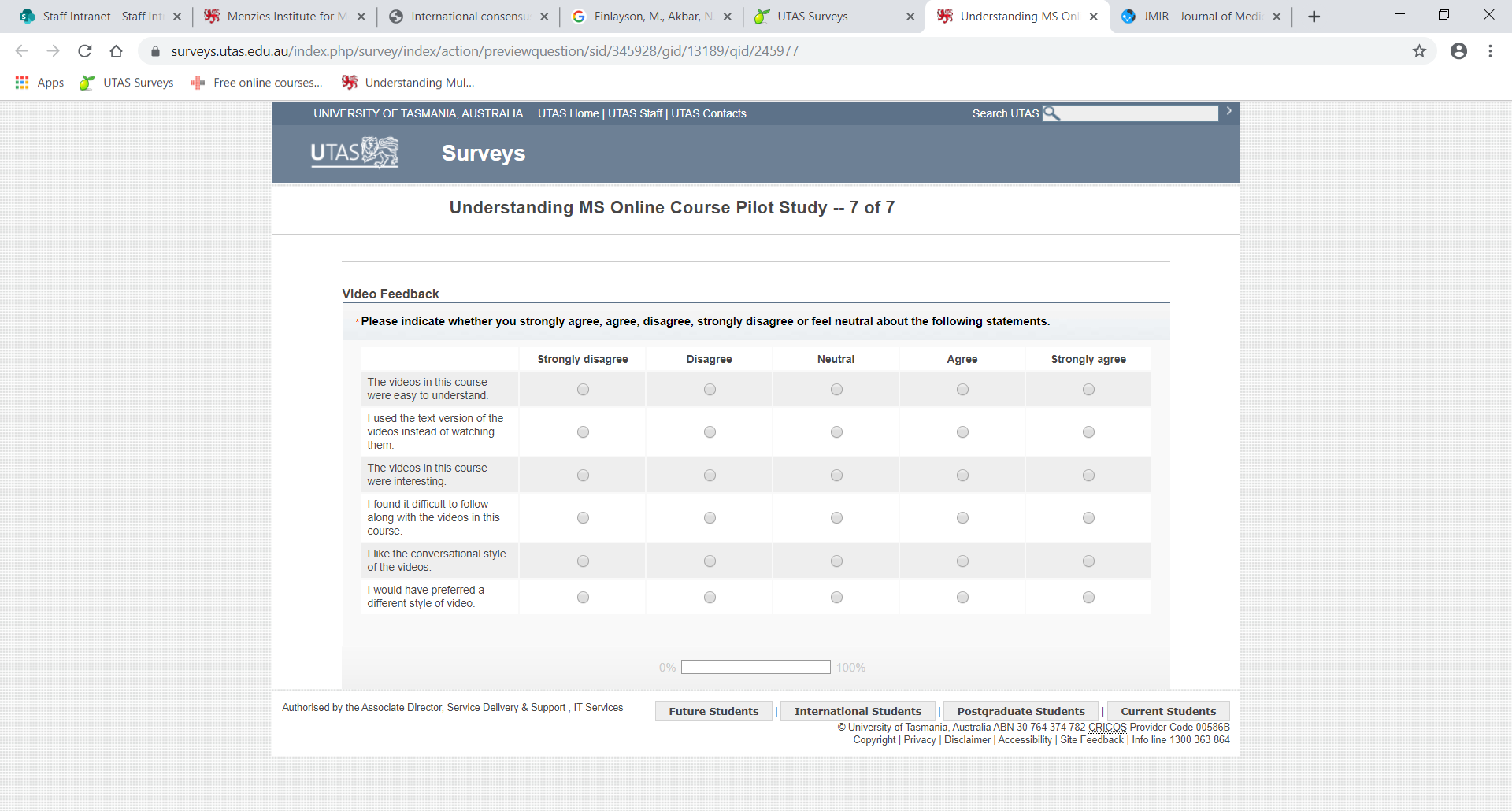


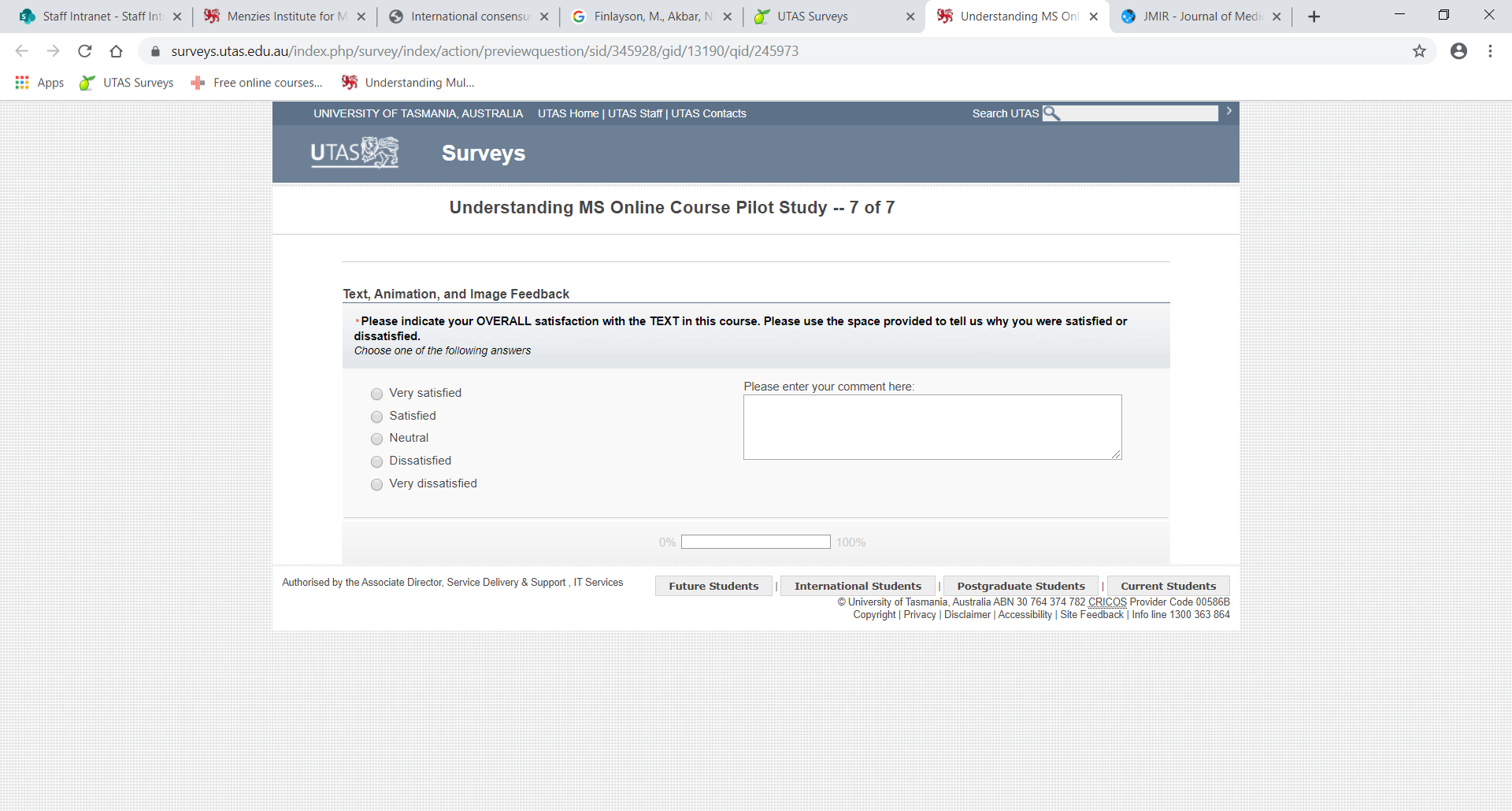


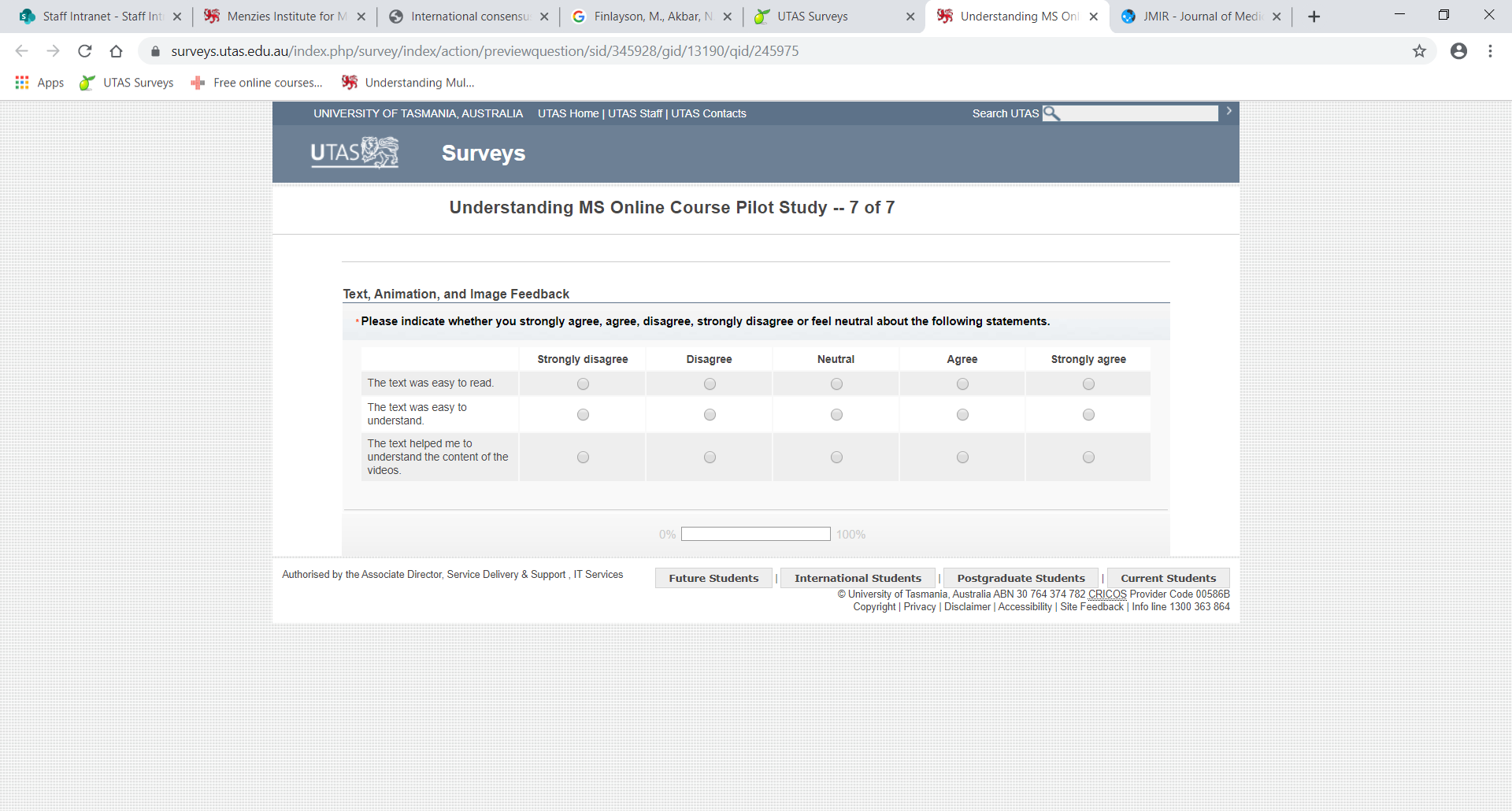


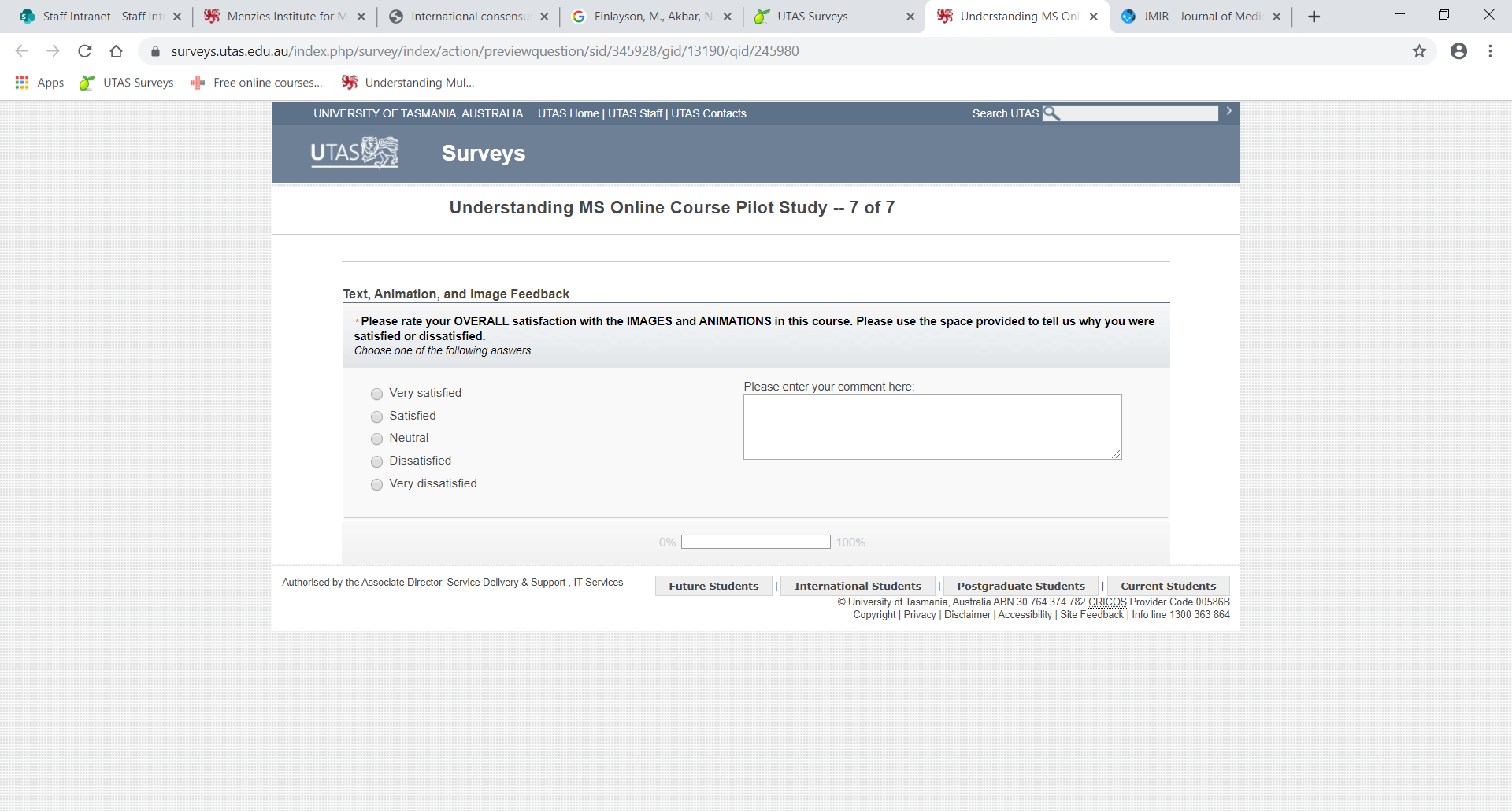


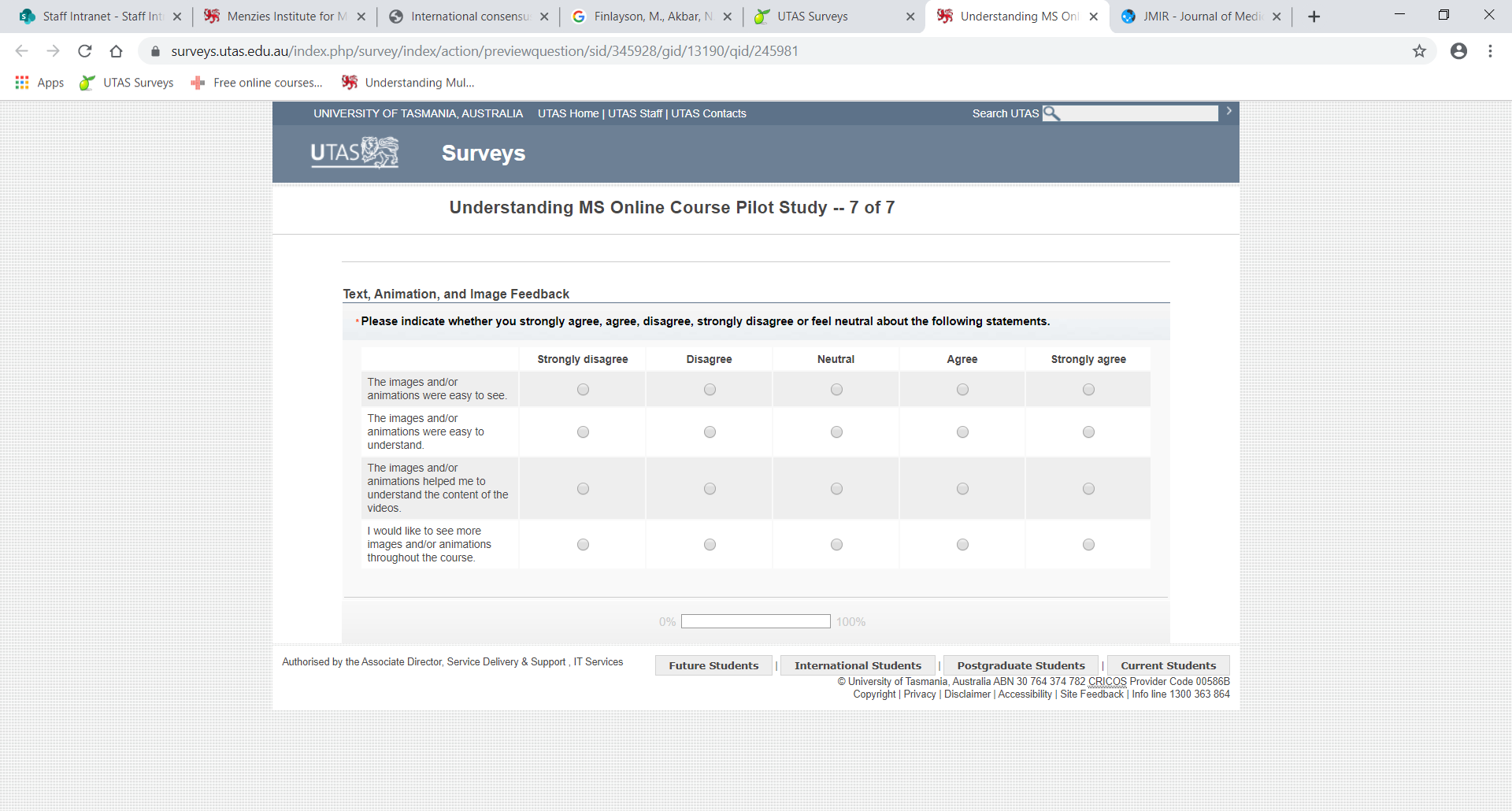


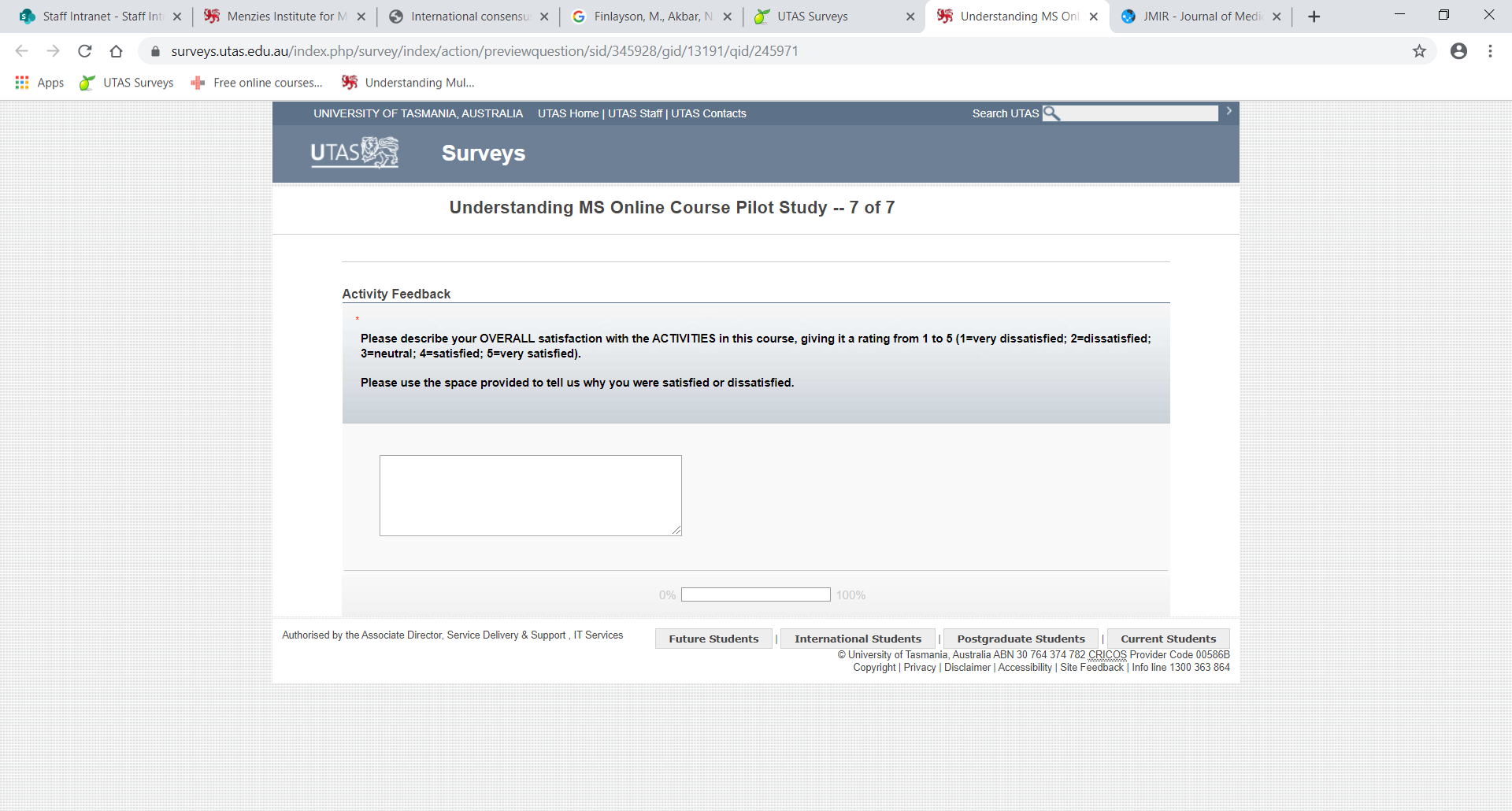


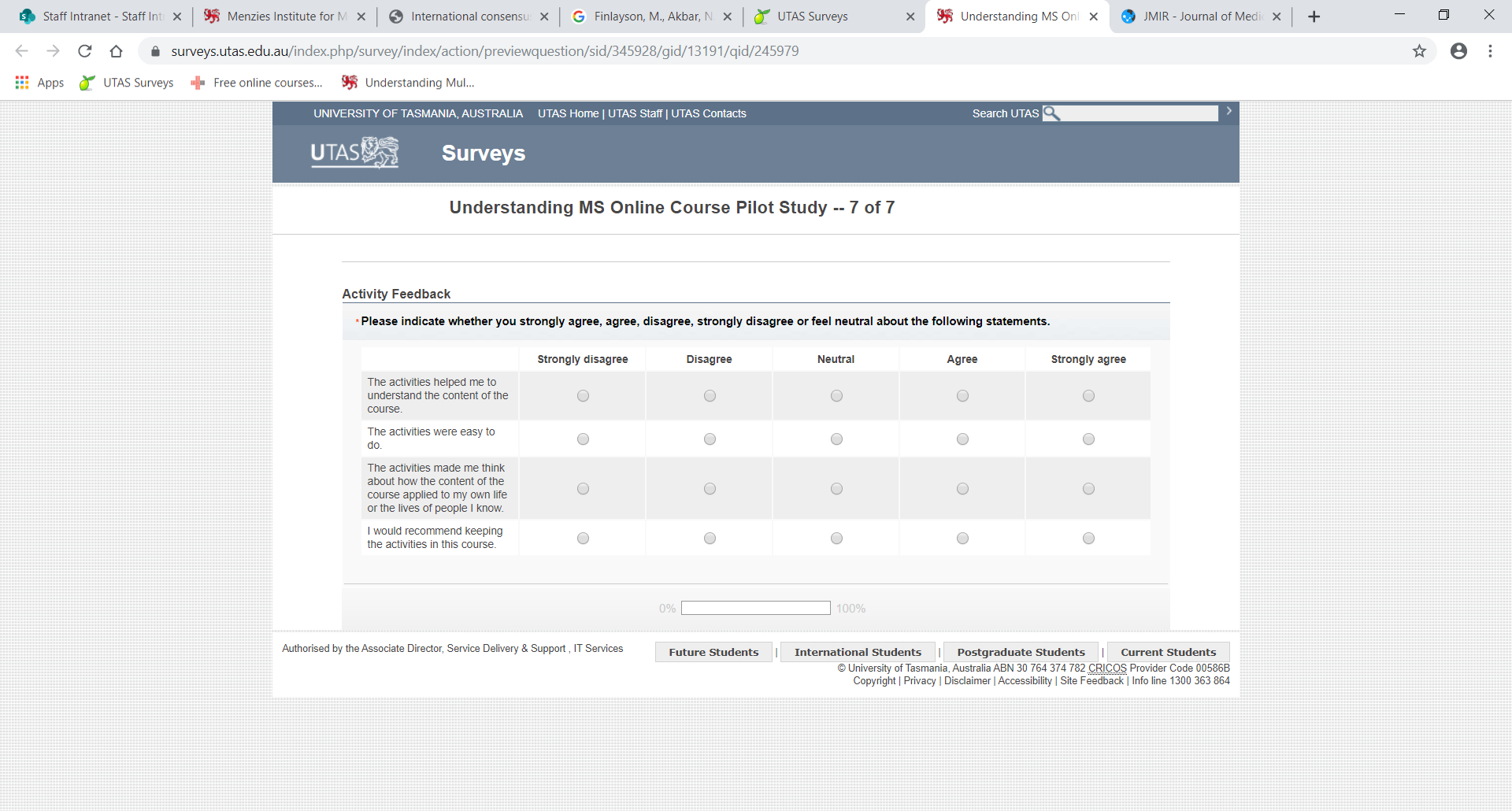


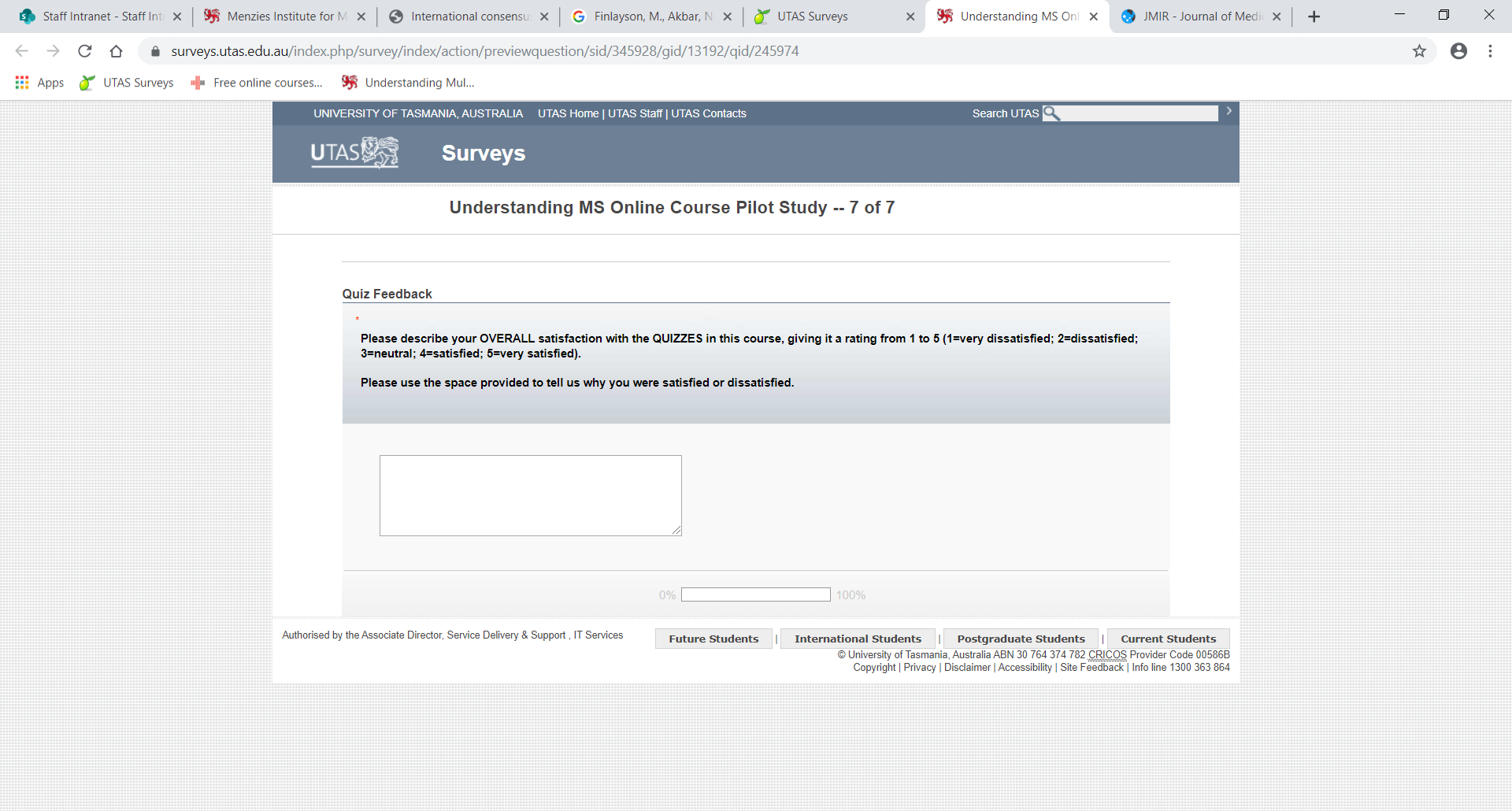


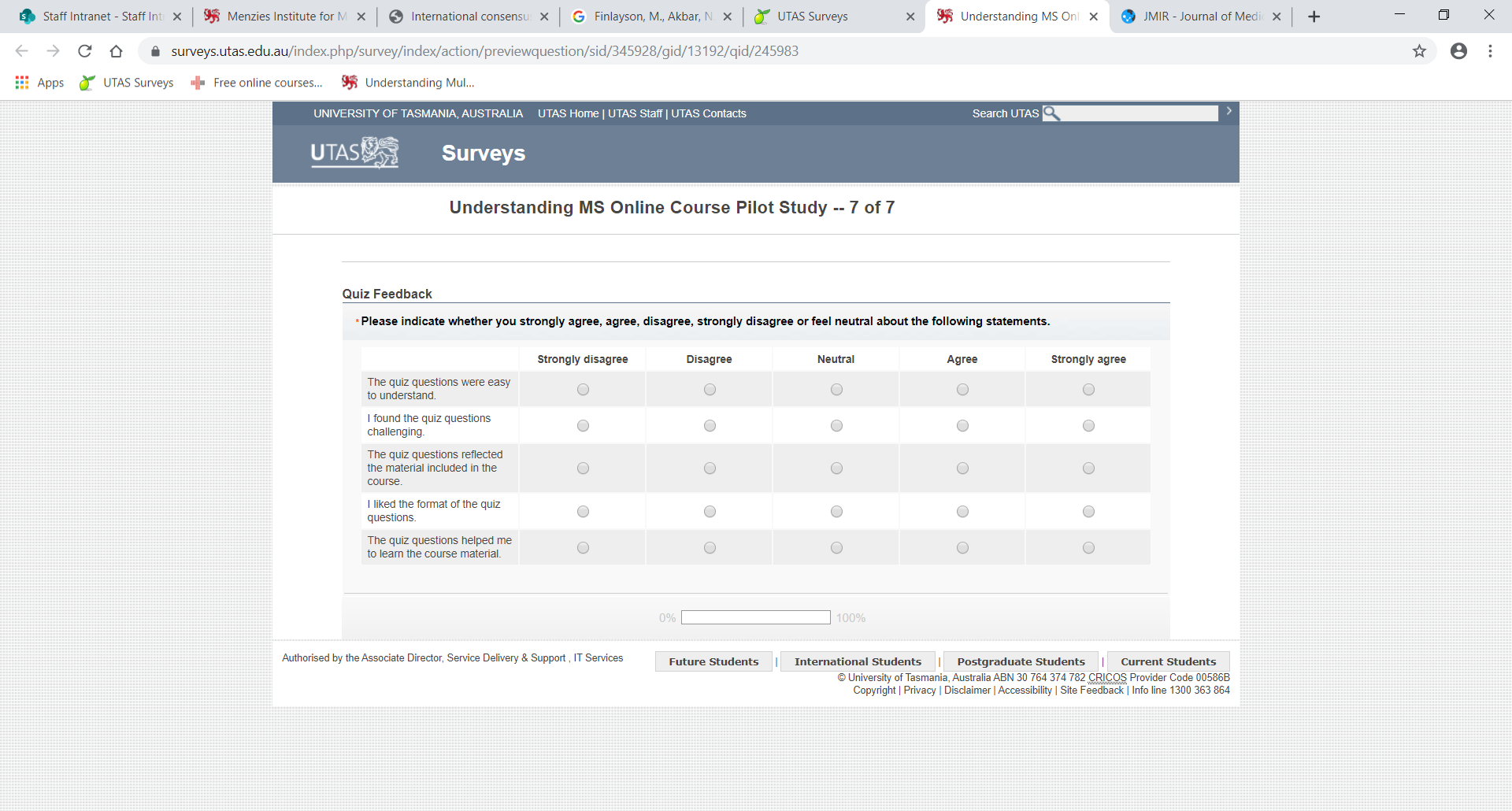


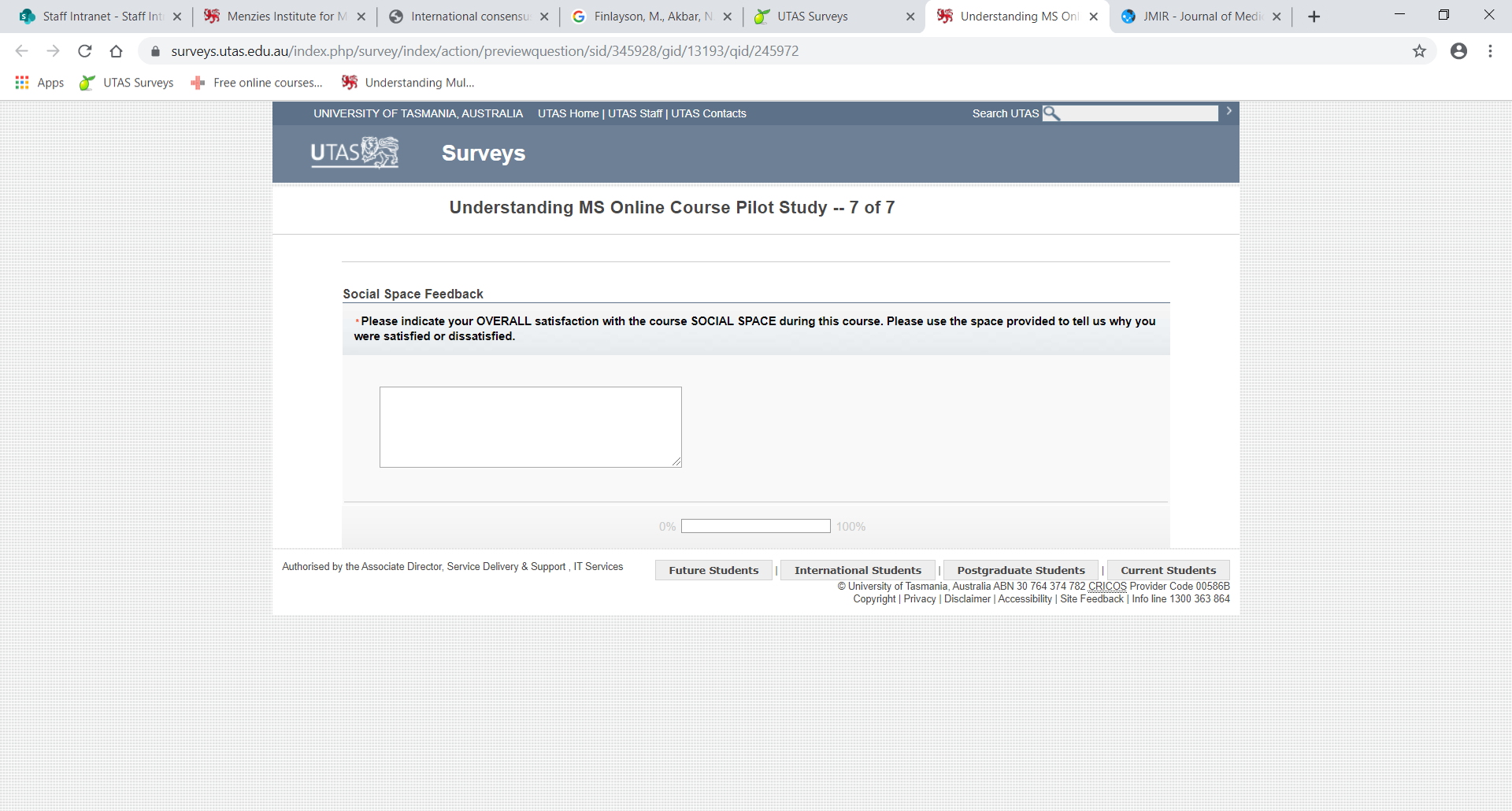


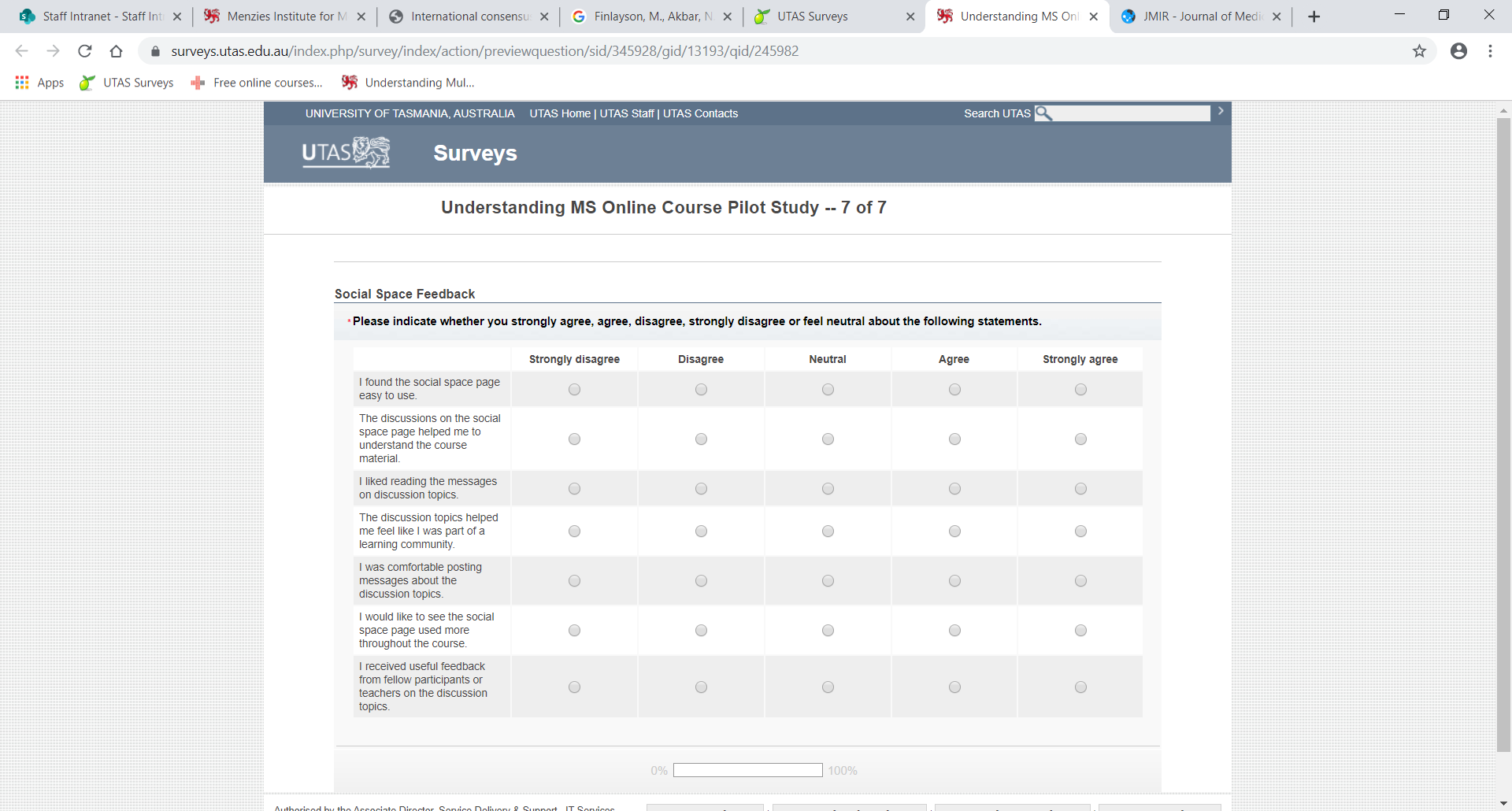


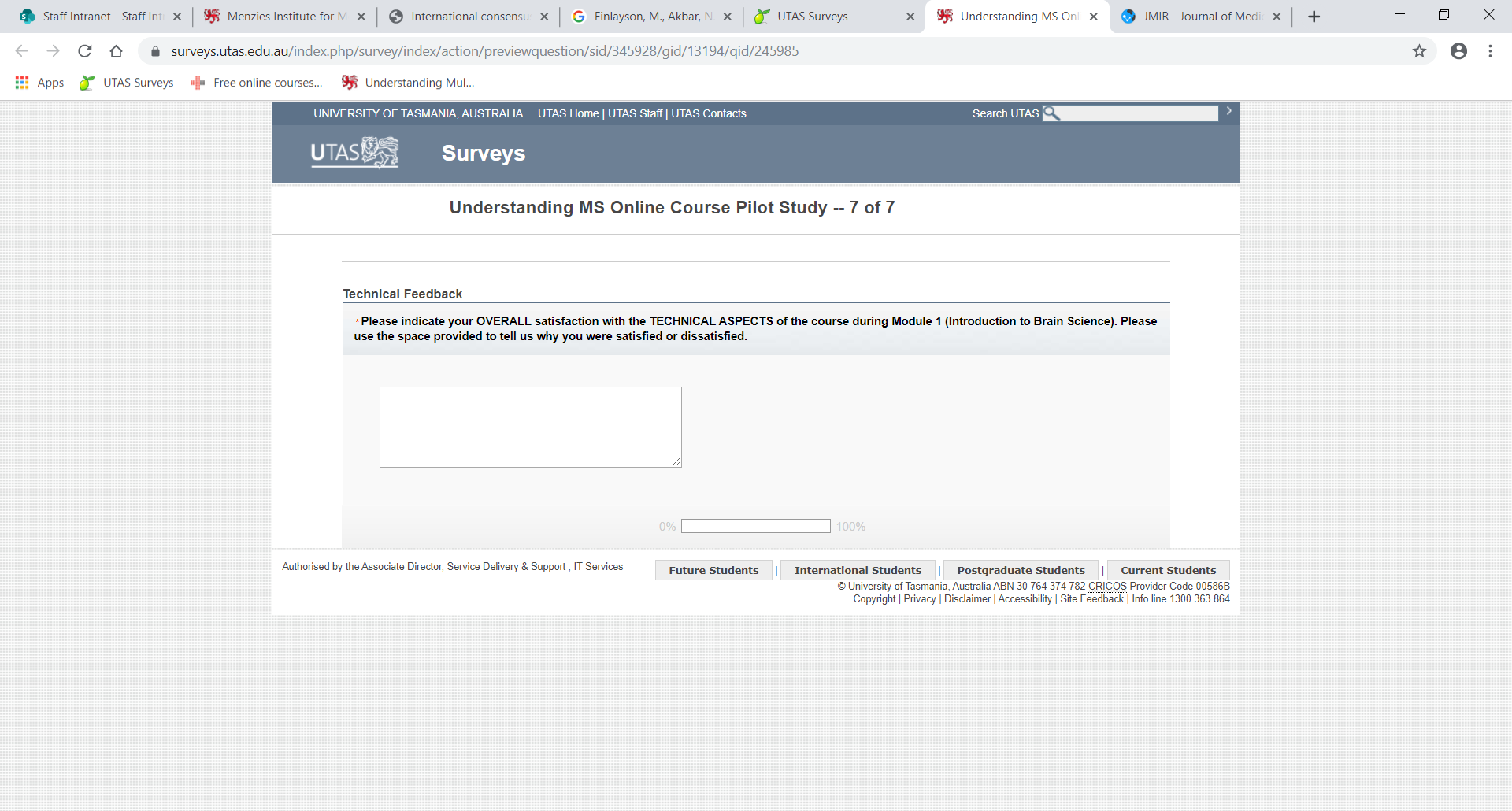


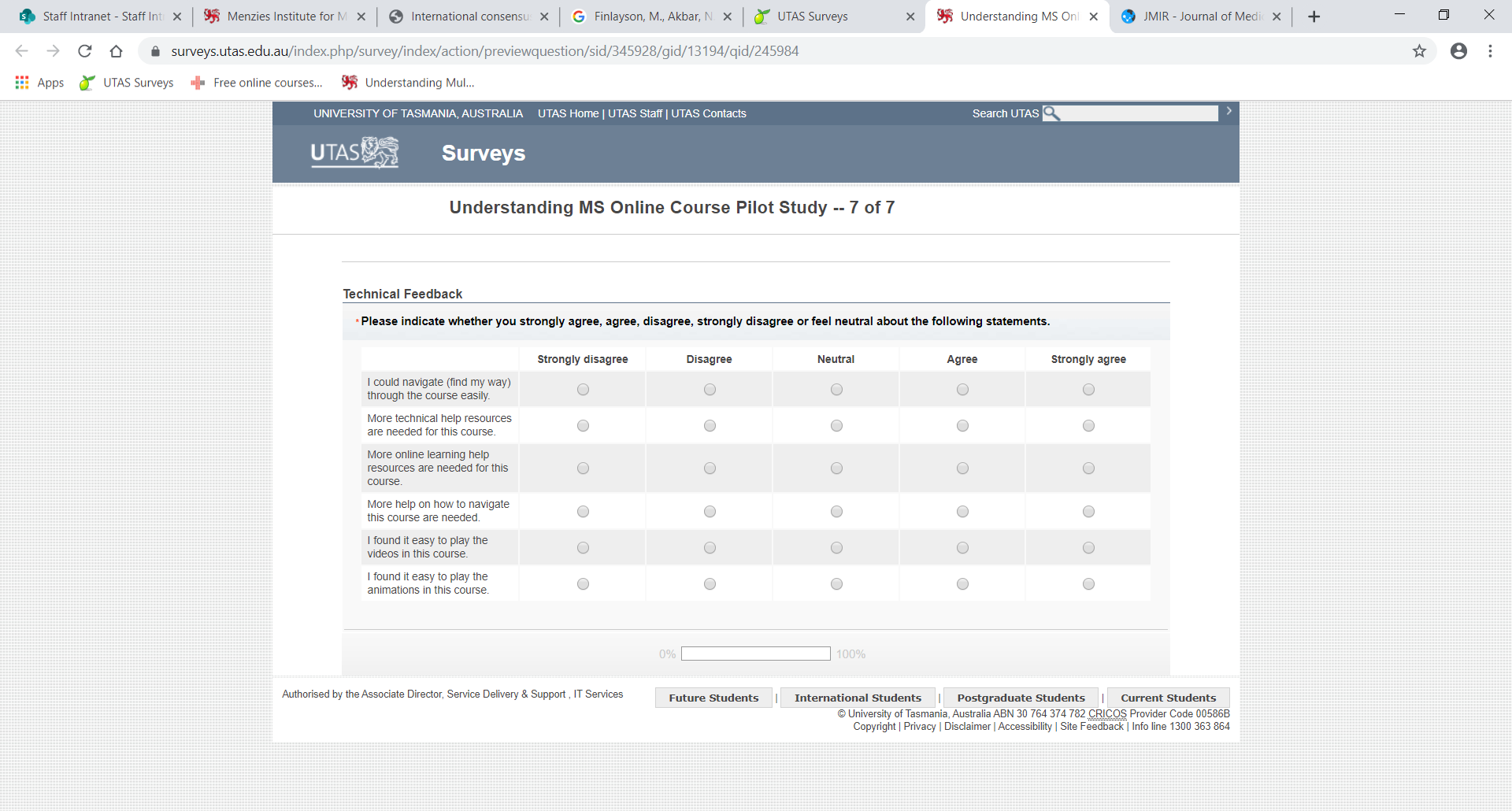


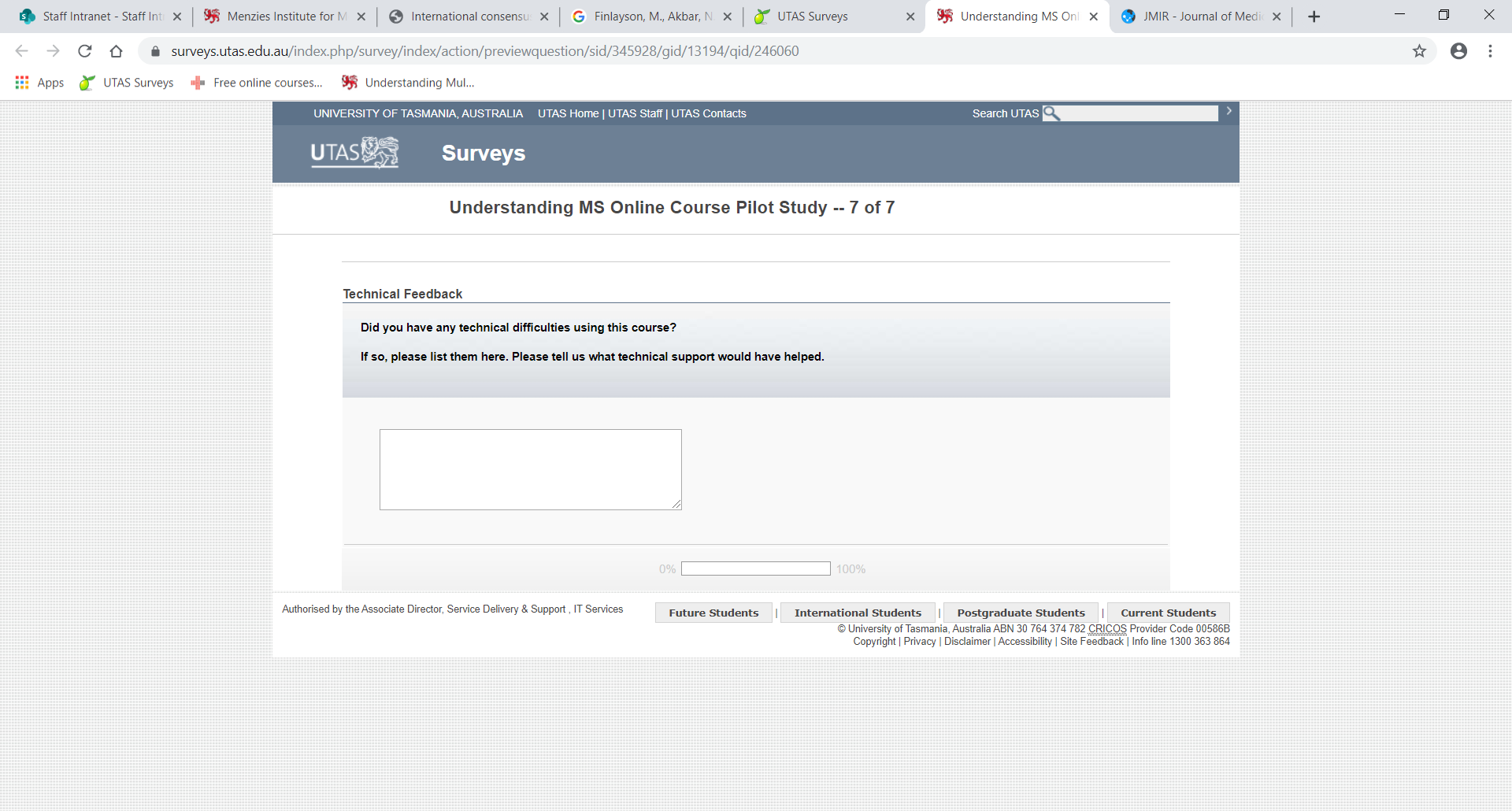


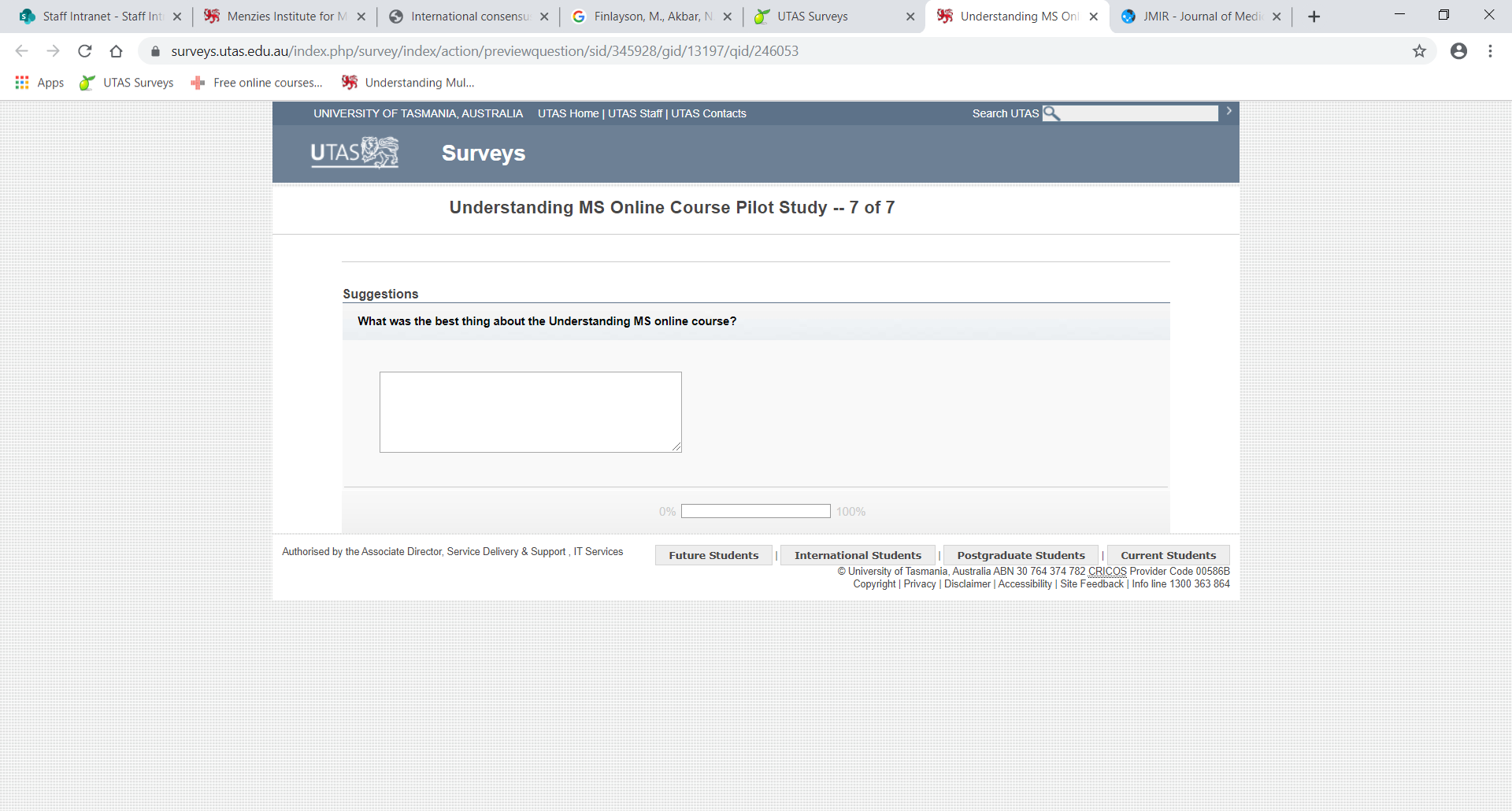


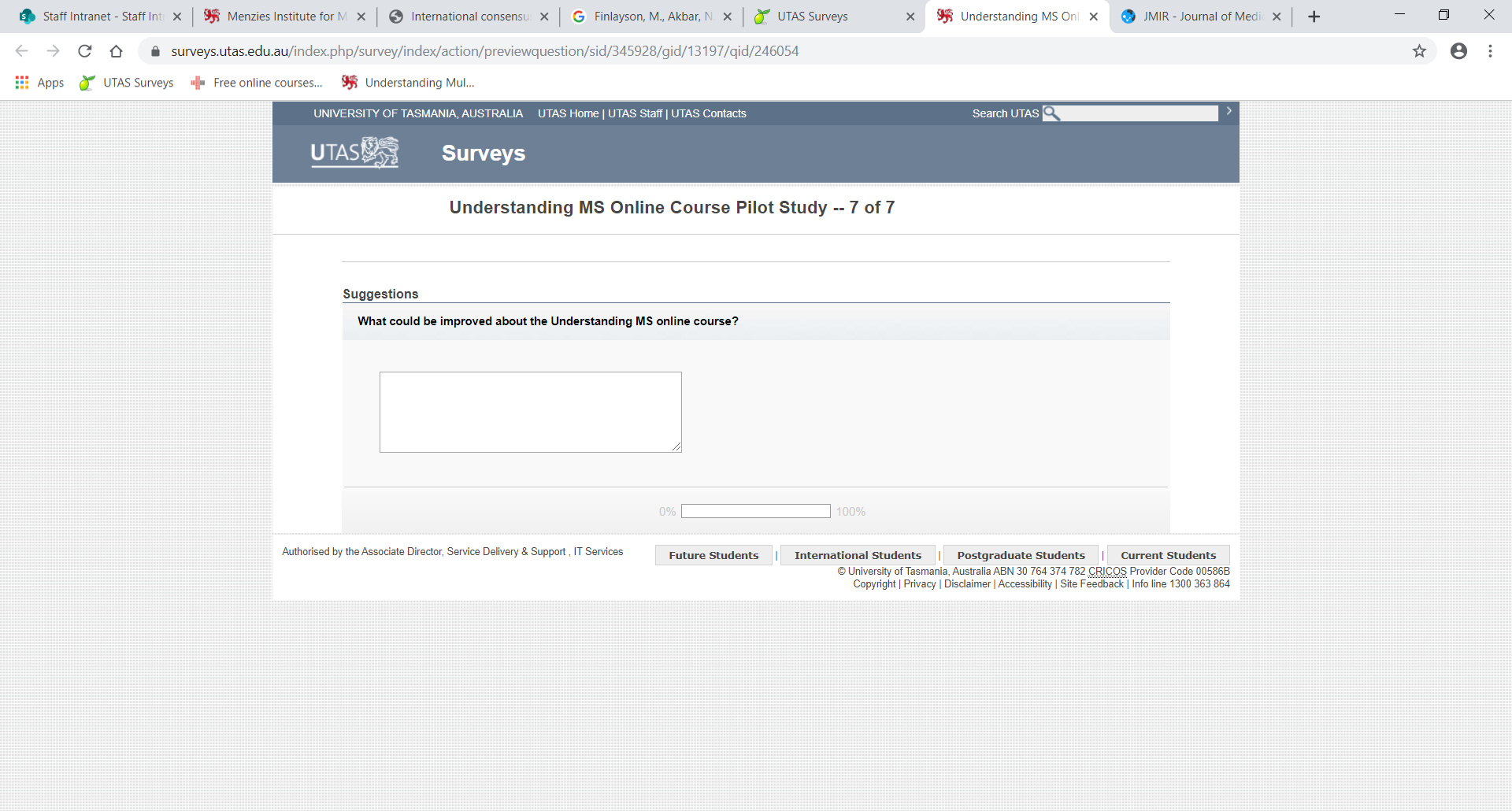


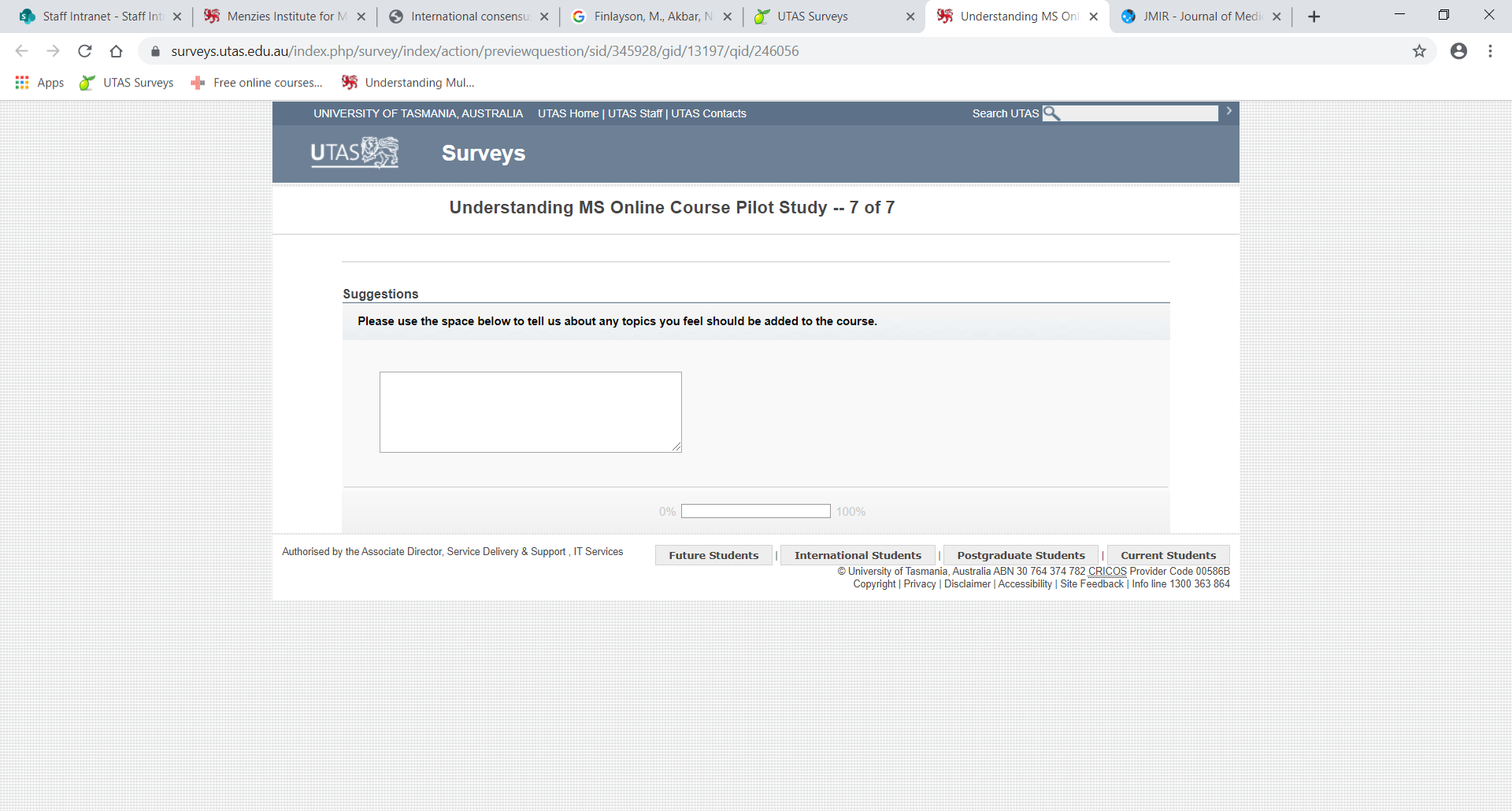

Supplement: Multimedia Appendix 6 [file jmir_v22i7e16687_app6.docx]

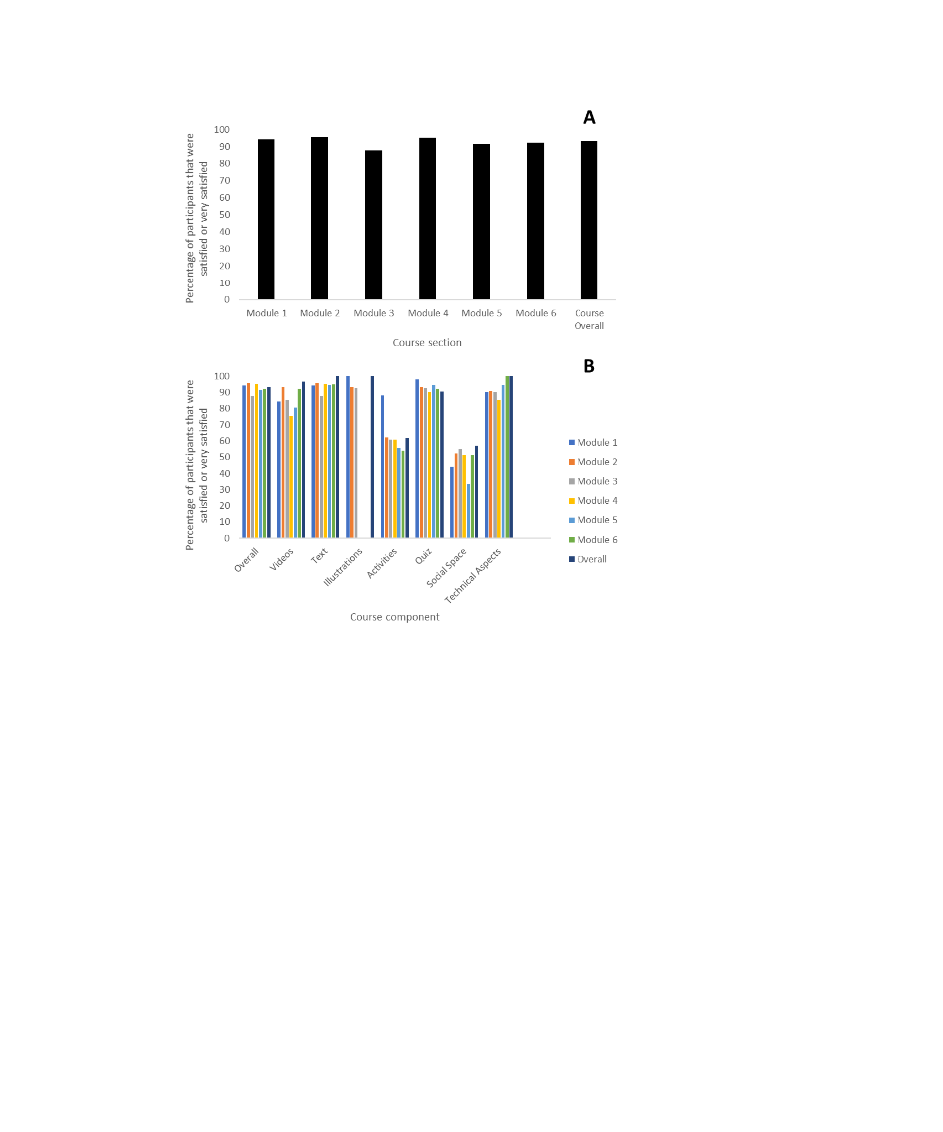

Supplement: Multimedia Appendix 7 [file jmir_v22i7e16687_app7.png]
