# Supplementary material for: Building a Successful Massive Open Online Course About Multiple Sclerosis: A Process Description
Source: J Med Internet Res. 2020 Jul 29;22(7):e16687. doi: 10.2196/16687 (PMC7424472; doi:10.2196/16687)
Supplement: Multimedia Appendix 4 [file jmir_v22i7e16687_app4.docx]

**Appendix 4:** Example quiz. Correct answers are bolded.

1. For every 1 man living with MS, there are ____ women living with MS.
2. 0
3. **3**
4. 9
5. 18
6. MS has a latitudinal gradient, meaning that people living __________ the North and South poles are more likely to develop MS than people who live __________ the equator.
7. **Close to; close to**
8. Far from; far from
9. Any distance from; any distance from
10. It is not clear how geographic location affects the risk of developing MS
11. What shapes MS demographics (patterns in the MS community described with statistics)?
12. Chance
13. Luck
14. **Risk**
15. It is not clear what shapes MS demographics
16. Individual risk is
17. The risk for a particular person
18. Is the result of the unique combination of risk factors a particular person has been exposed to throughout their life
19. Is made up of genetic, environmental and behavioural risk factors
20. **All of the above**
21. It is possible for scientists and health care providers to calculate individual risk
22. True
23. **False**
24. Population risk is
25. The average risk of a group of people
26. Scientists’ and health care providers’ best estimate for the risk for any particular person within that population
27. Is made up of genetic, environmental and behavioural risk factors
28. **All of the above**
29. Sometimes, despite a person who has tried to avoid all the sources of risk that they can (e.g. having a healthy diet, exercising regularly, not smoking, etc.) will still have a major health event, such as a heart attack. Why is that?
30. No one knows why this happens
31. **They are an outlier, because their individual risk was much HIGHER than the population risk**
32. They are an outlier, because their individual risk was much LOWER than the population risk
33. They did not exercise enough
34. In our hypothetical study, the relative risk of developing MS was 4 times greater in the high pie group compared to the low pie group. If this were true, what would it mean?
35. That we should never eat pie again
36. That this was a poor-quality study
37. **That the high pie group is more likely to develop MS than the low pie group. To understand the risk overall, we need to look at the raw numbers**
38. The relative risk is a meaningless result
39. Scientists use research studies to do all of the following EXCEPT:
40. Measure risk
41. **Disprove ideas that they don’t like**
42. Gather evidence for a particular idea so that they can be confident that is correct
43. Develop a greater understanding
44. When you are deciding whether or not an online source is reliable, you should ask all of the following questions EXCEPT:
45. Who wrote it?
46. What is the tone?
47. **Do I like what it says?**
48. When was it published?
