# Supplementary material for: Building a Successful Massive Open Online Course About Multiple Sclerosis: A Process Description
Source: J Med Internet Res. 2020 Jul 29;22(7):e16687. doi: 10.2196/16687 (PMC7424472; doi:10.2196/16687)
Supplement: Multimedia Appendix 5 [file jmir_v22i7e16687_app5.docx]

**Appendix 5:** List of discussion prompts and activities from the first iteration of the Understanding MS online course.

**Introduction**

Discussion prompt: You will be invited to participate in a number of discussion forums throughout the course. First, we would like to invite you to introduce yourself to the rest of the Understanding MS community. Just post a quick hello and a short statement on the Social Space Discussion Board about why you are undertaking the Understanding MS course and/or share with us what you hope to learn during your time here.

**Module 1: Introduction to Brain Science**

Activity: Body Central (interactive educational game about brain anatomy)

Discussion prompt: Putting knowledge into practice – In this module, you have learned about the underlying biology of MS. You have learned about the central nervous system an how it is affected by MS. How might this knowledge be helpful to you? How might you use the information from this module in conversations about MS?

**Module 2: Symptoms and Diagnosis**

Activity: Symptom Tracking – In this module, we discussed the symptoms of MS. We learned that they can change over time. This can make them challenging to track and then discuss at a later time, such as an appointment with a health care provider. One tool that can help is a symptom log or symptom tracker. Pick a symptom (it can be of MS or another condition) or symptoms that you might like to discuss with your doctor. **If you do not live with a chronic disease, feel free to track things like back pain, head aches or even hiccups.** Practice monitoring the symptom(s) over the next week using the included symptom log or one of the symptom tracker apps in the Additional Resources section on the following page.

Discussion prompt: MS symptoms & diagnosis—In this section, you have seen people living with MS, an MS nurse and a neurologist speaking about their experiences with MS symptoms. In this discussion board, we invite you to discuss the invisible symptoms of MS (e.g. fatigue) and why these symptoms might be particularly challenging for people living with MS and the MS community.

**Module 3: Demographics and Introduction to Risk**

Activity: Find an MS article and determine the quality of evidence – For this activity we ask that you perform an internet search to find a recent (published within the last two years) research article about MS. It can be from any source: a news site, a science site, an MS organisation site, etc. Please post a link to the article in this discussion board. Along with the link, please write a sentence or two describing the research in the article, if you think the article is reliable and why or why not.

Discussion prompt 1: Finding good sources – The internet is full of information, but not all of it is reliable. It is important to find sources of information that you can trust. Where do you usually go to find information about MS?

Discussion prompt 2: Symptom tracking follow-up – Last week, you were asked to track a symptom of MS or another condition using a symptom log or tracker app. How did the assignment go? Was keeping a log helpful? Did you notice anything you had not noticed before? Do you think it might be helpful for future appointments with health care professionals?

**Module 4: Risk Factors**

Activity: Goal Setting – None of us is perfect. Can you work to reduce your risk of a major health event? Please set a goal related to risk reduction using what you have learned about setting effective goals. Be sure that it is a SMART (specific, measurable, achievable, relevant, and timely) goal. Keep track of your progress using the activity log below, or another goal tracker log or app. We will follow-up on this activity in the next two weeks of the course.

Discussion prompt: What are some of the things that help you to reach the goals you set? – In this section, we learned about how to set effective goals. But setting the goal is just the first step. What are some of the things that have helped you achieve goals you have set in the past? Examples could be things like setting an alarm on your phone to remind you to take a vitamin or having an exercise buddy who keeps you accountable.

**Module 5: Disease Management**

Activity: Design a disease management plan – You are designing a disease management plan. It could be for MS or another condition, for you or for someone else. Who would you want to be involved in the plan? Some options might be: person living with MS, neurologist, physiotherapist, psychologist, family, and friends. Why do you want them to be involved? Can you identify people in your own life that you might ask to be involved? Please take a few minutes to reflect on these questions. You can enter your answer in the ‘Your Notes’ field below or write them down on a piece of paper.

Discussion prompt 1: Disease management plan – In this module, we have learned about the different parts of a disease management plan. What do you think are key parts of a disease management plan? Why is having a disease management plan important?

Discussion prompt 2: Goal tracking follow-up – Last week, you were asked to set a goal and track your progress using a goal tracker log or app. How is it going so far? Was it difficult to set a goal? Has tracking your progress been helpful?

**Module Six: Living with MS**

Activity: End of course goal setting – Set a goal or goals around how to use the information from this course. It could be to improve communication around MS, changing a behavior, setting up a disease management plan or something else. Be sure to use what you have learned about effective goal setting.

Discussion prompt 1: Talking about MS – In this section, we learned some strategies for communicating about MS. What do you think of these suggestions? What are some of the challenges you have encountered when communicating about MS? What are some of your best tips and tricks for communicating effectively about MS?

Discussion prompt 2: Goal tracking follow-up – In Module 4, you were asked to set a goal and track your progress using a goal tracker log or app. How is it going so far? Has tracking your progress been helpful? Do you think you will continue to track it?

**Completion**

Discussion prompt: Your course feedback – If you would like to provide any additional feedback or reflections on the course with the Understanding MS team and your fellow course participants, then please post it on the Your Course Feedback discussion forum. We would like to hear any feedback you consider important about any aspect of the course, to help us keep improving our MS education.
